# Intervention to Increase Condom Use Among Users of Sexually Transmitted Infection (STI) Self-Sampling Websites (Wrapped): Feasibility Randomized Controlled Trial

**DOI:** 10.2196/71611

**Published:** 2025-08-15

**Authors:** Katie Newby, Kayleigh Kwah, Lauren Schumacher, Rik Crutzen, Louise L Jackson, Stephen Bremner, Julia V Bailey, Katherine E Brown

**Affiliations:** 1 Public Health and Applied Behaviour Change (PHAB) Lab Centre for Research in Psychology and Sports University of Hertfordshire Hatfield United Kingdom; 2 Department of Health Promotion Care and Public Health Research Institute (CAPHRI) Maastricht University Maastricht The Netherlands; 3 Health Economics Unit Department of Applied Health Sciences University of Birmingham Birmingham United Kingdom; 4 Department of Primary Care and Public Health Brighton and Sussex Medical School Brighton United Kingdom; 5 eHealth Unit Department of Primary Care and Population Health University College London London United Kingdom

**Keywords:** sexual health, sexually transmitted infections, STIs, self-testing, condom, young people, adolescent, digital health, feasibility randomized controlled trial, eHealth

## Abstract

**Background:**

Sexually transmitted infections (STIs) such as chlamydia are common among young people and can lead to serious health issues if untreated. Although condoms are recommended for prevention, many young people report inconsistent use during penetrative sex. Web-based STI testing is becoming increasingly popular, but these services typically offer minimal support or guidance on preventing future infections. The “Wrapped” intervention aims to help young users of web-based STI testing use condoms consistently and correctly during penetrative sex, thus reducing future STI incidence.

**Objective:**

This study aims to assess whether and how it is possible to conduct a future randomized controlled trial (RCT) of the Wrapped intervention.

**Methods:**

Users of web-based STI testing aged 16 years to 24 years were randomized to an online, double-blind, 2-arm, parallel-group feasibility RCT in which Wrapped plus usual care (basic information on STIs and condom use) was tested against usual care alone. Main outcome measures were the proportion of the sampling pool recruited and return of valid chlamydia self-samples at month (M)12. Other outcome measures included return of valid chlamydia self-samples at M3; online survey completion at baseline, M3, M6, and M12; follow-up by demographic characteristics; and acceptability of intervention and measures.

**Results:**

Over 31weeks, 173 participants were recruited and provided a baseline chlamydia test result, representing 1.5% of the sampling pool (173/11,413; intervention: n=84; control: n=89). A valid chlamydia self-sample was returned by 75.7% (131/173; 95% CI 68.6-81.9) at M12. Therefore, 3574 participants, derived from a sampling pool of 238,266 service users, were estimated to be necessary to power a future full trial. Return of other follow-up measures included 75.1% (130/173) valid M3 chlamydia self-samples, 91.3% (158/173) M3 survey, 90.8% (157/173) M6 survey, and 90.8% (159/173) M12 survey. Participants at M12 appeared to broadly represent individuals in the sampling pool with some exceptions: a tendency for over-representation of participants who were older (20-24 years), of Black ethnicity, and in the least deprived quintile and under-representation of participants who were younger (16-19 years), male, and in deprivation quintile three. There was some evidence that attrition was patterned by ethnicity and age in ways that compounded initial recruitment patterns. Drop-out attrition was evident, with retention higher at M12 for the intervention group (72/84, 86%) than the control group (59/89, 66%). Eleven adverse events relating to participation were reported. A priori criteria for success were met.

**Conclusions:**

A full trial is feasible. Although the recruitment rate was low, the high volume of young people using web-based STI testing services (approximately 585,000 annually based on the latest data) provides a sufficient pool to meet the required sample size. To ensure balanced representation, strategies to address potential under- and over-representation of certain demographic subgroups by M12 should be implemented.

**Trial Registration:**

ISRCTN Registry ISRCTN17478654; http://www.isrctn.com/ISRCTN17478654

**International Registered Report Identifier (IRRID):**

RR2-10.2196/43645

## Introduction

Young people are disproportionately affected by acute sexually transmitted infections (STIs) like chlamydia [1]. The risk of contracting STIs is not, however, evenly distributed, with higher rates observed among individuals who are Black; reside in areas of greater deprivation; or are gay, bisexual, and other men who have sex with men (GBMSM) [1]. If left untreated, STIs can lead to severe health issues such as pelvic inflammatory disease and infertility [2]. Additionally, they can negatively impact quality of life and are associated with stigma, which may discourage individuals from seeking care and contribute to further transmission [2]. Although condoms for penetrative sex are advised for STI prevention, young people frequently report inconsistent use [3].

To maximize accessibility, the National Institute for Health and Care Excellence (NICE) recommends that a range of settings are provided for STI testing including web-based resources [4]. There has been rapid growth in the use of web-based STI testing services over the last 5 years [5]. STI self-sampling websites are as effective as in-person services at reaching groups with higher STI diagnosis rates [1]. Repeated use of web-based testing services is common, however, with chlamydia positivity remaining high for those who re-test, suggesting that using online testing services does not lead to future preventative behavior. Although national STI management standards state that all people accessing STI testing should be provided with health promotion interventions to prevent future infection (eg, encouraging condom use) [6], most STI testing websites do not comply with this [7]. Given the continued growth of the web-based STI testing sector, there is an urgent need to address this.

Evidence from meta-reviews suggests that behavioral interventions have modest favorable effects on condom use and STI incidence, including for different populations and when delivered via face-to-face or digital methods [8,9]. However, although most included studies use the most defensible type of design, that is randomized controlled trials (RCTs) or quasi-experimental studies, these are generally of low quality, with infrequent use of objective, biological outcomes and a tendency for short durations of follow-up [10,11]. Accordingly, there is an urgent need for well-designed, high-quality trials to generate robust evidence on the impact of behavior change interventions on sexual health outcomes.

“Wrapped” is a multicomponent, interactive digital intervention aiming to support young people to use condoms correctly every time they have penetrative sex [12]. It was developed especially for users of web-based STI testing services aged 16 years to 24 years. Content addresses important behavioral determinants of condom use [13-16], namely condom use attitudes, condom availability, and self-efficacy for condom use and communication. Wrapped is now ready to be definitively tested using an RCT to assess its effectiveness and cost-effectiveness. To prepare for this RCT, a feasibility RCT (fRCT) was conducted to determine whether and how it might be possible to carry out the trial.

## Methods

### Reporting Standards

See Multimedia Appendix 1 for the CONSORT (Consolidated Standards of Reporting Trials) pilot and feasibility trials extension checklist [17].

### Study Design

This was a 2-arm, parallel-group fRCT comparing Wrapped plus usual care (basic information on STIs and condom use) with usual care alone (control). The rationale, design, and methods have been previously described [18]. In preparation for the fRCT, a separate study was undertaken to develop a participant recruitment and retention strategy [19]. A process evaluation was also undertaken, the findings of which can be found in the full report provided to the funder [20].

### Public and Patient Involvement

Study development and delivery were supported by 6 users of a web-based STI self-sampling service (Freetest.me; operated by Preventx Ltd) aged 20 years to 24 years old (2 women, 4 men). This included drafting all participant-facing materials and iteratively testing fRCT web-based surveys to maximize content validity, readability, clarity, and comprehensiveness.

### Governance and Registration

Sponsorship was provided by the University of Hertfordshire. Study oversight was provided by an independent Data Monitoring and Ethics Committee (DMEC) and a Study Steering Committee (SSC). The study was registered as International Standard Randomized Controlled Trial Number (ISRCTN) 17478654 [21]. Transparent reporting of study protocol changes can be found on the funder’s website [22].

### Ethical Considerations

Ethical approval was granted by NHS East Midlands - Leicester Central Research Ethics Committee (reference 20/EM/0275). The study was conducted according to the principles expressed in the declaration of Helsinki [23]. All participants provided informed consent prior to participation in the study. Only anonymous study data are reported in this paper and the supplementary material. Participants received up to £100 (US $135.48) in vouchers in return for participation in this study (see the “Participant Incentives” subsection for further information).

### Research Objectives

The primary objectives of the fRCT were to estimate the following parameters for planning a definitive RCT: (1) the rate of recruitment of eligible participants and (2) the rate of participant follow-up for the definitive RCT primary outcome measure (chlamydia positivity at 12 months measured using biological samples). Chlamydia positivity was chosen as the RCT primary outcome measure, as it is the most diagnosed STI among young people. It will therefore be most sensitive to any intervention effect.

The secondary objectives for the fRCT were as follows: (1) estimate the rate of participant follow-up for the definitive RCT secondary outcome measure chlamydia cumulative incidence; (2) identify whether the level of deprivation of the final sample is representative of web-based STI self-sampling users; (3) identify whether differential retention occurs across groups (gender, ethnicity, sexual identity, deprivation, randomized groups, chlamydia diagnosis at baseline); (4) estimate chlamydia positivity at 12 months in the intervention and control groups (to support sample size calculation for the definitive RCT); (5) estimate the rate of attrition at 3 months, 6 months, and 12 months and ways of minimizing this; (6) determine the feasibility and participant acceptability of all primary and secondary outcome measures (including health economic); (7) identify which recruitment message(s) results in the highest rate of recruitment; (8) identify costs and resource use associated with the intervention for health care services and users (to inform the design of a future definitive RCT with an economic evaluation); (9) measure contamination of intervention effect in the control group; and (10) identify possible adverse effects of the intervention.

For reasons of parsimony, findings in relation to a further objective “Identify and remove intervention friction points to minimize attrition and maximize future intervention dose” are reported elsewhere [24].

### Progression Criteria

The following a priori progression criteria were agreed by the SSC and DMEC: (1) proportion of Preventx users recruited to the feasibility trial sufficient to obtain the sample size required for the definitive RCT; (2) 60% of participants followed up for the definitive RCT primary outcome measure at 12 months; (3) index of multiple deprivation (IMD) quintile distribution for the final sample comparable with that of individuals in the sampling pool; and (4) adverse events judged as sufficiently infrequent or serious to cause concern.

### Participant Timeline

Recruitment took place between March 2021 and October 2021. Each participant was invited to complete research activities over a 12-month period with data collection completed in October 2022. Table 1 sets out the nature and timing of these activities.

**Table 1:** Participant activities and timing for the Wrapped feasibility randomized controlled trial.

Activity	Month 0	Month 3	Month 6	Month 12
Read participant information	✓	—^a^	—	—
Complete consent	✓	—	—	—
Complete survey	✓	✓	✓	✓
Self-report chlamydia test result	✓	—	—	—
Complete chlamydia self-sample	—	✓	—	✓

^a^Not applicable.

### Participant Incentives

In line with the protocol, participants initially received up to £65 (US $88.06) in vouchers for the completion of all research activities. This was, however, later increased to £85 (US $115.16) and then again to £100 (US $135.48) to stimulate recruitment. See Multimedia Appendix 2 for the timing of these increases and amount paid per research activity.

### Participants and Study Setting

Young people requesting a test for chlamydia via a web-based STI self-sampling service, namely Freetest.me or SH.UK (both operated by Preventx Ltd), were invited to participate in the study. Preventx provides an STI self-sampling service to 70 local authority areas across England (free at the point of use for most young people). Users residing in 1 of 5 geographical areas (selected to represent English demography), namely East Sussex, Kingston-Upon-Thames, Northamptonshire, Somerset, and Warwickshire, were invited to participate.

Regarding the inclusion criteria, individuals were eligible for participation if they were aged 16 years to 24 years, a user of either freetest.me or SH.UK, and living in one of the aforementioned 5 geographical areas involved in the study.

Recruitment was initially limited to the freetest.me service and 4 geographical areas. Use of SH.UK and the expansion of geographical areas were in response to contractual changes between Preventx Ltd and the first cohort of local commissioning areas; one contract came to an end before recruitment began (meaning we could no longer recruit individuals from that area; this location was replaced by 2 new areas), and 3 areas migrated from freetest.me to SH.UK delivery.

Regarding the exclusion criteria, individuals with no internet access, who reported having sexual preferences that meant that they were unlikely to have penetrative sex (penis in vagina or anus) over the 12-month data collection period, or who did not feel able to fully commit to the study were ineligible.

Users of freetest.me and SH.UK were invited to participate using an advertisement placed on the “thank you” page of each website, which was viewed by service users upon placing an order for a home STI self-sampling kit. Only those aged 16 years to 24 years and living in one of the 5 participating local authority areas were shown an advertisement (the sampling pool). Seven advertisements, presenting different value propositions (each highlighting a combination of altruistic and financial incentives of participation), were displayed on rotation. Of these advertisements, 6 were co-developed with the Public and Patient Involvement (PPI) group, and one was suggested by a study co-investigator (see development of our recruitment and retention strategy for detail on how these advertisements were selected [19]). This set of advertisements was edited during recruitment to reflect the increasing incentive amount being offered. A call to action for 16- to 19-year-olds was also added to one advertisement type to assess the effect on recruitment of making a direct appeal to younger participants. A hyperlink embedded within the advertisement took users to Research Electronic Data Capture (REDCap), a secure data capture and management platform, for participant information and consent.

### Sample Size Calculation

In line with recommendations [25], the fRCT aimed to have primary outcome data for 60 participants per group to enable design parameters for a full trial to be estimated with good precision. This was inflated to allow for an estimated 25% nonreturn of the initial chlamydia self-test sample (based on statistics provided by Preventx; 60/0.75=80) and a further estimated 30% drop-out by 12 months (80/0.7=114 per group).

### Control Condition

Typically, STI self-sampling websites provide their users with basic health promotion material on STIs and condom use. To replicate this level of usual care, a standalone website was created [26] and presented to participants randomized to the control condition.

### Intervention

The Wrapped intervention includes a website, along with condoms and other products dispatched through orders made via the site. A detailed description of intervention content and development, including screenshots and example videos and images of intervention materials, is described elsewhere [12]. An overview is presented in Table 2.

**Table 2:** Overview of Wrapped intervention content as presented to participants in the Wrapped feasibility randomized controlled trial.

Component	Frequency of access	Description
Condom sample pack	One-off order	A box of 12 condoms and 2 sachets of lubricant; users instructed via leaflet provided to test condoms (solo practice: take on/off; attend to smell, texture, fit/feel; masturbate and focus on pleasurable sensations) to identify preferred type(s) and develop positive associations with condoms
Order condoms	Limited to one order per month	Users given access to discreet condom ordering service providing bundles of 6 or 12 free condoms of their choice (from a selection of 12 types) plus 3 sachets of lubricant (from a selection of 2 types)
Condom carrier	One-off order	Users given a free carrier (small faux leather wallet; choice of 2 designs) with discreet pocket for storing condoms when “out and about”; note: the original carrier design (headphone case) as described by Newby and colleagues [12] was changed to the wallet design based on consultation with our PPI^a^ group.
Using condoms	Unlimited access	A video made by young people giving step-by-step instructions on how to put on a condom, including tips on how to do this in a way that is pleasurable for self and partner
Discussing condoms	Unlimited access	A series of 7 videos made by young people who give ideas on how to communicate with a partner about condoms; they talk about what has and hasn’t worked for them and how to cope with resistance.
Real life	Unlimited access	A series of 3 videos featuring real couples talking positively about using condoms during sex; shots of talking are interspersed with scenes of kissing, touching; and sex with condoms (no genitals or penetration shown); aims to increase positive associations with condoms; available to ≥18 years old only
Information on STIs^b^ and condom use	Unlimited access	This replicates the usual care information provided via the comparator website.

^a^PPI: Public and Patient Involvement.

^b^STIs: sexually transmitted infections.

Intervention content is tailored to the user according to their responses to questions presented to them at registration that identify their main barriers to condom use (see Multimedia Appendix 3 for details). All users receive the condom sample pack and the information on STIs and condom use as standard. The other components are added in line with their expressed need.

### Randomization and Blinding

Participants were randomized to the control or intervention group (1:1 allocation) within REDCap on completion of the baseline (M0) survey. This was an automated process with no human involvement. Stratification across groups, using randomly permuted blocks, (ethnicity, sexual identity, deprivation) was performed to balance participants across the trial groups. Prior to randomization, manual checks were performed to establish that each individual was a unique person and a genuine user of freetest.me or SH.UK and had answered the baseline survey with reasonable care. Those not meeting these criteria were not randomized.

A hyperlink sent by email (triggered on completion of randomization) directed participants to the intervention or comparator website. Both websites had responsive designs, meaning that the display of content was optimized according to the device used to access it (desktop, tablet, mobile phone). Participants in the intervention condition were required to create an account and to sign-in on each subsequent access; this was not a requirement of the control website. Participant IDs were appended to the URL used by each participant to access their assigned website. Checks were made to ensure that website visitors were only those with an assigned ID. All participants were free to interact at any time with the respective materials. Participants, Preventx Ltd laboratory staff performing STI testing and reporting results, and those assessing outcomes were all blind to allocation.

### Measures

#### Survey Completion

All survey data were collected via REDCap. The baseline survey collected information on contact details (including postcode used to identify IMD quintile), demographic characteristics (age, sex, gender identity, ethnicity, sexual identity), recent STI diagnosis, STI treatment, sexual well-being, condom use, condom use intentions, multilevel behavioral determinants targeted by the intervention, health-related quality-of-life (using the EQ5D-5L and the Short Form 12 [SF-12]) [27,28], and resource use (health care, public sector, private sector) and costs. Except for contact details and demographic characteristics, all items were repeated within the surveys at months 3 (M3), 6 (M6), and 12 (M12). All follow-up surveys included an additional item to record evidence of adverse events. The 12-month survey also included items to identify evidence of contamination between groups. If no response was received, 3 SMS text messages, followed by 1 email then 1 telephone call, were used to prompt completion. Participants not providing any data 20 days after the initial survey was sent out were classed as nonresponders. Surveys continued to be sent to nonresponders at subsequent time points unless a participant asked to be withdrawn from the study.

#### Chlamydia Testing

At 10 days after randomization, an SMS text message was sent to participants containing a link to a single-item survey (on REDCap) to self-report the result of their baseline chlamydia test. Participants were asked to record the result and any treatment received if applicable. Participants were able to trigger a repeat of this message up to two times if their STI self-sampling kit had not yet been received nor returned. Nonresponders were sent a single SMS text message reminder. Those not providing these data 4 weeks after the first SMS text message was sent were classed as nonresponders.

To measure chlamydia infection at months 3 and 12, the research team sent chlamydia self-sampling kits directly to participants. The kits contained the usual user instructions for collecting a sample and returning it by Freepost to Preventx for processing. If no sample was received, up to 3 SMS text messages, followed by 1 email then 1 phone call, were used to prompt return. Nonresponders were categorized as those who had not returned their kit 30 days after it was posted out. Except for participants who asked to be withdrawn from the study, all nonresponders were sent test kits at subsequent time points. An SMS text message containing a link to a survey was sent to participants with a positive test result 2 weeks postresult to record whether infection had been treated. If the participant indicated that treatment was incomplete or they did not respond, they were sent 1 reminder SMS text message to obtain this information. Local NHS trusts were informed of positive test results so they could provide appropriate treatment and follow-up care.

### Analysis

Data were downloaded from REDCap, and analysis was performed using SPSS v28 (IBM Corp) [29]. Planned descriptive analyses are presented in the following sections.

#### Primary Outcome Measures

The primary outcome measures included the percentage of participants recruited to the fRCT (assessed as the number of participants randomized) of those in the sampling pool (those shown the study advertisement via freetest.me or SH.UK) and the percentage of participants with a valid primary outcome measure (chlamydia positivity measured using biological samples) at 12 months.

#### Secondary Outcome Measures

The secondary objectives were the (1) percentage of participants with outcome data required to measure cumulative incidence of chlamydia (measured using M3 and M12 chlamydia self-samples and the relevant items within M3, M6, and M12 surveys); (2) distribution of IMD quintiles among those in the sampling pool compared with that of the final sample; (3) percentage of participants with a valid primary outcome measure at 12 months by group (gender, ethnicity, sexual identity, deprivation, randomized groups, chlamydia diagnosis at baseline); (4) percentage of participants with a positive chlamydia test result at 12 months in the intervention and control groups; (5) attrition curves comparing the percentage of participants in the trial at M3, M6, and M12 plotted for the intervention and control groups (drop-out attrition); (6) percentage completion of survey items required to measure primary and secondary outcomes in the main trial (including self-report of chlamydia result at baseline, results from biological samples, demographic information, self-report of condom use, and data needed for cost-effectiveness and cost utility analyses); (7) percentage of participants randomized as a direct result of each of the different advertisements used; (8) completeness of data on costs and resource use that would be needed for the economic evaluation in the definitive RCT; (9) percentage of participants in the control group who reported (at M12) any exposure to Wrapped; and (10) percentage of participants who reported (at M12) having experienced an adverse event during the study.

## Results

### Participants

**Figure 1:**
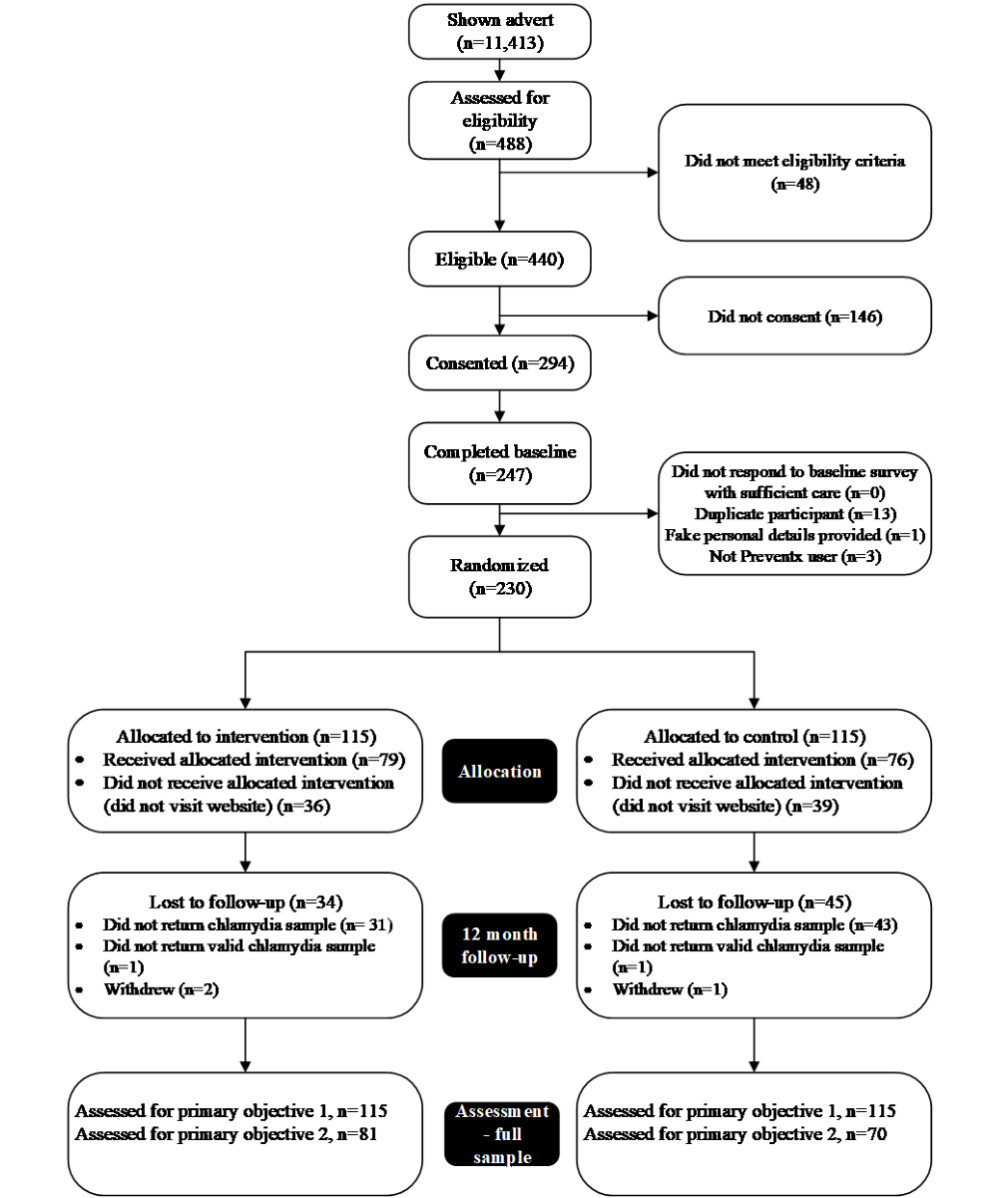
Participant flow diagram for the Wrapped feasibility randomized controlled trial.

Over the 31-week recruitment period, a total of 11,413 individuals were shown the study advertisement, of which 488 went on to complete the eligibility questions. The eligibility criteria were not met by 48 (9.8%) of the 488 individuals who completed the eligibility questions. Of those eligible, 294 individuals provided consent. Of those consenting, 247 (247/294, 84%) completed the baseline survey, 17 of whom were excluded because there was evidence of duplicate participation (duplicate personal details provided; n=13), the individual not being a Preventx user (duplicate Preventx user ID; n=3), or fake personal details provided (address not valid; n=1). The remaining 230 individuals were randomized; 115 participants were allocated to the intervention, and 115 were allocated to the control. Figure 1 shows the flow of participants through the study in line with CONSORT pilot and feasibility trials reporting standards [17].

### Withdrawals

Three participants, 2 in the intervention group and 1 in the control group, withdrew from the study. None of these individuals requested that the data they had provided up to that point be withdrawn.

### Reported Sample

Unless otherwise stated, the sample reported on in this paper is a subsample (n=173) of all randomized participants (n=230). This subsample includes only randomized participants who reported (1) their baseline chlamydia test result and (2) if positive, that they took the full course of prescribed antibiotics (indicating they were likely infection free at baseline). This subsample is reported here as it provides a better approximation of the nature of the sample in the full trial for whom data on baseline chlamydia positivity (primary outcome) are required, both to ensure balance across groups and to calculate chlamydia cumulative incidence (secondary outcome; randomized participants must be infection free at baseline). Reporting on the subsample was approved by our DMEC, SSC, funder, and NHS research ethics. Note that the same analyses were also performed for the full sample; this can be found in the full report provided to the funder [27].

### Demographics of the Sample

The demographic characteristics of participants at baseline are presented in Table 3.

**Table 3:** Demographic characteristics overall and by trial group at baseline for participants in the Wrapped feasibility randomized controlled trial

Characteristic		Intervention (n=84)	Control (n=89)	Total (n=173)
**Age (years), n (%)**				
	16	1 (1.2)	2 (2.2)	3 (1.7)
	17	1 (1.2)	1 (1.1)	2 (1.2)
	18	3 (3.6)	6 (6.7)	9 (5.2)
	19	3 (3.6)	4 (4.5)	7 (4)
	20	11 (13.1)	19 (21.3)	30 (17.3)
	21	21 (25)	9 (10.1)	30 (17.3)
	22	9 (10.7)	18 (20.2)	27 (15.6)
	23	15 (17.9)	17 (19.1)	32 (18.5)
	24	20 (23.8)	13 (14.6)	33 (19.1)
Age (years), median (IQR)		22 (2)	22 (3)	22 (3)
**Age group (years), n (%)**				
	16-19	8 (9.5)	13 (14.6)	21 (12.1)
	20-24	76 (90.5)	76 (85.4)	152 (87.9)
**Ethnicity (stratification factor), n (%)**				
	White	73 (86.9)	76 (85.4)	149 (86.1)
	Mixed or multiple ethnic groups	2 (2.4)	6 (6.7)	8 (4.6)
	Asian or Asian British	1 (1.2)	4 (4.5)	5 (2.9)
	Black, African, Caribbean, or Black British	7 (8.3)	3 (3.4)	10 (5.8)
	Other ethnic group	1 (1.2)	0	1 (0.6)
**Gender, n (%)**				
	Female	56 (66.7)	72 (80.9)	128 (74)
	Male	24 (28.6)	16 (18)	40 (23.1)
	Nonbinary or gender fluid	3 (3.6)	1 (1.1)	4 (2.3)
	Other	1 (1.2)	0 (0)	1 (0.6)
**Gender not as assigned at birth, n (%)**		3 (3.6)	3 (3.4)	6 (3.5)
**Sexual identity^a^ (stratification factor), n (%)**				
	Heterosexual	58 (69)	56 (62.9)	114 (65.9)
	Gay	8 (9.5)	3 (3.4)	11 (6.4)
	Bisexual	16 (19)	29 (32.6)	45 (26)
	Other	2 (2.4)	1 (1.1)	3 (1.7)
**Financial situation while growing up (stratification factor), n (%)**				
	Very comfortable - I had everything I needed and more	7 (8.3)	7 (7.9)	14 (18.1)
	Comfortable - money was never an issue	18 (21.4)	20 (22.5)	38 (22)
	Fairly comfortable but it was necessary to keep an eye on money	27 (32.1)	27 (30.3)	54 (31.2)
	Things were ok but money was tight sometimes	19 (22.6)	21 (23.6)	40 (23.1)
	Money seemed to be a problem a lot of the time	9 (10.7)	10 (11.2)	19 (11)
	It was always a struggle to get the basics (food, clothes, heating)	4 (4.8)	4 (4.5)	8 (4.6)
**Index of multiple deprivation quintile, n (%)**				
	Quintile 1 (most deprived)	11 (13.1)	14 (15.7)	25 (14.5)
	Quintile 2	14 (16.7)	13 (14.6)	27 (15.6)
	Quintile 3	18 (21.4)	26 (29.2)	44 (25.4)
	Quintile 4	21 (25)	23 (25.8)	44 (25.4)
	Quintile 5 (least deprived)	20 (23.8)	13 (14.6)	33 (19.1)
**Relationship status, n (%)**				
	Single	34 (40.5)	35 (39.8)	69 (40.1)
	In a casual relationship with 1 person	18 (21.4)	15 (17)	33 (19.2)
	In casual sexual relationships with 2 or more people	9 (10.7)	9 (10.2)	18 (10.5)
	In a serious relationship with 1 person	22 (26.2)	24 (27.3)	46 (26.7)
	In a serious relationship with more than 1 person	0 (0)	0 (0)	0 (0)
	Combining serious and casual relationships	1 (1.2)	4 (4.5)	5 (2.9)
	Other	0 (0)	1 (1.1)	1 (0.6)
	Missing	0 (0)	1 (1.1)	1 (0.6)

^a^No participants self-reported their sexual identity as lesbian or asexual.

### Primary Objectives

#### Research Objective 1: Estimate the Rate of Recruitment of Eligible Participants

Over the 31-week (217 days) recruitment period, 173 participants (the subsample) were recruited to the study at a recruitment rate of 0.80 participants per day (173/217), representing 1.5% of the sampling pool (173/11,413).

#### Research Objective 2: Estimate the Rate of Participant Follow-Up for the Definitive RCT Primary Outcome Measure (Chlamydia Positivity at 12 Months Measured Using Biological Samples)

Of the baseline sample, 75.7% (131/173; 95% CI 68.6-81.9) of participants completed and returned a valid chlamydia self-sample at M12.

### Secondary Objectives

#### Research Objective 3: Estimate the Rate of Participant Follow-Up for the Definitive RCT Secondary Outcome Measure Chlamydia Cumulative Incidence

Data on chlamydia test positivity provided at any point during the 12-month study period (as identified using M3 or M12 chlamydia self-samples or relevant items within the M3, M6, or M12 surveys) would be used in the full trial to calculate chlamydia cumulative incidence. Of the total sample, 65.9% (114/173) completed all these measures.

#### Research Objective 4: Identify Whether the Level of Deprivation of the Final Sample is Representative of Online STI Self-Sampling Users

Table 4 presents the proportion of individuals in IMD quintiles 1 (most deprived) to 5 (least deprived) in the sampling pool and at M12.

**Table 4:** Distribution of index of multiple deprivation (IMD) quintiles among service users in the sampling pool and participants in the study at the 12-month (M12) follow-up for the Wrapped feasibility randomized controlled trial.

IMD quintile	Sampling pool (n=11,413), n (%)	M12 follow-up sample (n=131), n (%)
1 (most deprived)	1418 (12.4)	21 (16)
2	2136 (18.7)	19 (14.5)
3	3110 (27.2)	29 (22.1)
4	2770 (24.3)	32 (24.4)
5 (least deprived)	1979 (17.3)	30 (22.9)

These data indicated that the M12 sample was broadly representative of the sampling pool across IMD quintiles with two exceptions. For IMD 3, it appeared that participants were less well represented at M12 than in the sampling pool. For IMD 5 (least deprived), there also appeared to be a higher relative proportion of participants at M12 than in the sampling pool.

For completeness, additional analysis was performed to examine whether the sample available for analysis represented the sampling pool across other demographic characteristics, namely gender, ethnicity, and age (see Table S1 in Multimedia Appendix 4 for findings; note: comparison of the M12 sample with the sampling pool was not possible for sexual identity as data on this characteristic were not available for the sampling pool).

#### Research Objective 5: Identify Whether Differential Retention Occurs Across Groups (Gender, Ethnicity, Deprivation, Sexual Identity, Randomized Groups, Chlamydia Diagnosis at Baseline)

To identify whether there was evidence of differential retention across demographic characteristics, the relative proportions of participants at baseline and M12 were compared across gender, ethnicity, deprivation, and sexual identity. For completeness, a comparison by age was made too, with participants grouped as 16-19 years and 20-24 years. Data are presented in Table 5. For gender, ethnicity, deprivation, and sexual identity, participants at M12 broadly represented those at baseline. A higher proportion of participants aged 20 years to 24 years and a lower proportion of participants aged 16 years to 19 years were observed in the final sample than at baseline. With respect to deprivation, as observed when comparing M12 with the sampling pool, when comparing M12 with baseline, there was evidence of a slight under-representation of IMD 3 and a slight over-representation of IMD 5.

**Table 5:** Distribution of gender, ethnicity, deprivation, sexual identity, and age among participants in the Wrapped feasibility randomized controlled trial at baseline and in the study at the 12-month follow-up (M12).

Characteristics		Baseline (n=173), n (%)	M12 follow-up (n=131), n (%)
**Gender**			
	Female	128 (74)	96 (73.3)
	Male	40 (23.1)	32 (24.4)
	Other^a^	5 (2.9)	3 (2.3)
**Ethnicity**			
	White	149 (86.1)	113 (86.3)
	Mixed or multiple ethnic groups	8 (4.6)	4 (3.1)
	Asian or Asian British	5 (2.9)	3 (2.3)
	Black, African, Caribbean, or Black British	10 (5.8)	10 (7.6)
	Other ethnic group	1 (0.6)	1 (0.8)
**IMD^b^ quintile**			
	1 (most deprived)	25 (14.5)	21 (16)
	2	27 (15.6)	19 (14.5)
	3	44 (25.4)	29 (22.1)
	4	44 (25.4)	32 (24.4)
	5 (least deprived)	33 (19.1)	30 (22.9)
**Sexual identity^c^**			
	Heterosexual	114 (65.9)	83 (62.9)
	Gay	11 (6.4)	10 (7.6)
	Bisexual	45 (26)	36 (27.5)
	Other	3 (1.7)	2 (1.5)
**Age (years)^d^**			
	16-19	21 (12.1)	11 (8.4)
	20-24	152 (87.9)	120 (91.6)

^a^Other includes transgender and nonbinary or fluid gender (data on these gender categories not reported by Preventx and therefore unavailable for comparison purposes).

^b^IMD: index of multiple deprivation.

^c^No participants self-reported their sexual identity as lesbian or asexual.

^d^The median age at both time points was 22 years

We also examined the proportion of participants in each of the randomized groups retained in the study at M12 (measured as the proportion of participants in the baseline sample who went on to provide a valid chlamydia self-sample at M12) and the retention of participants by chlamydia self-report at baseline (see Tables S2 and S3, respectively, in Multimedia Appendix 4). This showed that retention was higher for the intervention group (72/84, 86%) than the control group (59/89, 66%) and that retention among participants who reported positive and negative results at baseline was similar.

#### Research Objective 6: Measure Chlamydia Positivity at 12 Months in the Intervention and Control Groups (to Support Sample Size Calculation for the Definitive RCT)

Table 6 presents the data on test positivity at M12, broken down by intervention and control groups.

See Multimedia Appendix 5 for the trial sample size calculation.

**Table 6:** Chlamydia positivity at the 12-month follow-up (M12) for participants in the Wrapped feasibility randomized controlled trial

Restricted sample	Intervention	Control	Total
Sample, n	84	89	173
Positive cases reported, n	1	3	4
Test positivity	1.2	3.4	2.3

#### Research Objective 7: Identify the Rate of Attrition at 3 Months, 6 Months, and 12 Months and Ways of Minimizing This

Attrition was explored by examining the proportion of participants who completed each of the study measurements (see Table S4 in Multimedia Appendix 4). Overall, completion was higher for the follow-up surveys (90%-91%) than for the follow-up chlamydia self-sampling kits (75%-76%). Note that attrition is not presented using attrition curves as planned. Given that all participants were invited to complete all measures (regardless of prior completion), this skewed the presentation of results.

#### Research Objective 8: Determine the Feasibility and Participant Acceptability of all Primary and Secondary Outcome Measures (Including Health Economic)

All survey items had fully complete or near-complete data, with the latter defined as <5% missing data (see Table S5 in Multimedia Appendix 4).

#### Research Objective 9: Identify Which Recruitment Message(s) Results in the Highest Rate of Recruitment

Over the course of the recruitment period, 13 different advertisements were presented to freetest.me and SH.UK service users. See Table S6 in Multimedia Appendix 4 for the proportion of participants recruited with each message. There was no clear pattern in terms of which advertisement(s) resulted in higher recruitment.

#### Research Objective 10: Identify Costs and Resource Use Associated With the Intervention for Health Care Services and Users (to Inform the Design of the Definitive RCT and the Future Economic Evaluation)

Table 7 summarizes data collected on intervention costs during the trial. These data are illustrative only due to the small sample size. For details on how these costs were derived, see Multimedia Appendix 6.

**Table 7:** Summary of costs for intervention and control websites tested in the Wrapped feasibility randomized controlled trial.

Type of resource use	Total cost: intervention (£^a,b^)	Total cost: control (£^a,b^)
Condom sample packs	429.12	—^c^
Condom carriers	145.39	—
Condom ordering service	791.28	—
Website (maintenance and updating)	230.00	115.00
Total cost	1595.79	115.00
Average cost per participant (n=115 in each group)	13.88	1.00

^a^Costs are presented in £ as of 2021/2022.

^b^A currency exchange rate of £1=US $1.34 is applicable.

^c^Not applicable.

Costs and resource use were successfully collected and analyzed for all time points (baseline, M3, M6, and M12).

In Table S7 in Multimedia Appendix 4, details of health care service use at M12 are presented as an illustration of how the data would be used as part of an economic evaluation in a future definitive trial. The main health care resource use reported was related to sexual health clinics and general practitioner visits. A range of comprehensive cost data was collected; however, difficulties were experienced in capturing detail on some types of resource use, such as the length of time different contraceptives had been prescribed for.

#### Research Objective 11: Measure Contamination of Intervention Effect in the Control Group

Data on the full sample are presented here. Of all participants in the control group who completed the M12 follow-up survey (n=96), 42 (43%) reported exposure to Wrapped; that is, they indicated that they recognized one or more images from the intervention website (presented to them as screenshots within the M12 survey). This level of contamination was unexpected and investigated further. One possible route to contamination was a participant informing another person(s) about the study, this person then participating and being randomized into a different group of the study, followed by the 2 individuals sharing intervention content. Although there was some limited evidence of friendship pairs or groups participating in the study, there was no evidence of content sharing; examination of website log and data analytics records showed that the intervention website had only been accessed by those with valid participant numbers (and therefore that individuals in the control group had not gained access using rogue or fake numbers) and that repeat visits were only by those using the condom ordering component (indicating that the sharing of log-in details to allow others to gain access was unlikely). Further, although it was possible that participants in the intervention condition had shown the Wrapped website to others in the control group (that is, allowing them to physically view their screen), this seemed unlikely given that product ordering is restricted by design (either one per user or limited in quantity and frequency, meaning that others could not gain anything in this respect) and that there were few repeat views of videos. Given the limited evidence of contamination, the most likely explanation was that the question designed to detect this (in the M12 survey) was unreliable. Both the intervention and control websites had consistent branding (website name, logo, color palette, icons). It is possible that, when control group participants were asked if they recognized “some or all of the images” shown within 3 screenshots of the intervention website, they interpreted this literally (that is, they recognized the branding and therefore “some of the images”) or that the branding gave the screenshots a feeling of familiarity.

#### Research Objective 12: Identify Possible Adverse Effects of the Intervention

Participants were asked to report any adverse events related to their participation in the study using a fixed-response format in all follow-up surveys. Three options represented anticipated adverse events, with the final option for “other problem,” which if endorsed, routed participants to an open-ended question asking for further information. An open-ended text box for participants to provide further detail about the event and its impact was displayed when any of the fixed response options was selected.

Table S8 in Multimedia Appendix 4 presents the number of reported instances for each event type (collected at any time point) and any qualitative comments from the reporting participants received via survey or by email (in response to aftercare relating to the event). Most adverse events concerned disclosure of STI testing. Although 1 individual reported that participation in the study had led to an increase in their use of pornography, this participant was allocated to the control group (content thought to have the potential to lead to an increased use of pornography was on the Wrapped intervention website only). This individual was contacted and asked if they would be willing to provide further information, but none was received.

### Review of Progression Criteria

After completing the data analysis, separate meetings were held with the DMEC and SSC to assess the progression criteria. Prior to these meetings, a report detailing evidence for each criterion was distributed to members (see Multimedia Appendix 7). During the meetings, the evidence was presented, followed by a session of questions and discussion. A portion of the meeting was conducted in a closed format, without the research team, to allow members to deliberate in confidence. DMEC members voted during this closed session, while SSC members cast their votes afterward using a Microsoft form. Both groups were quorate and agreed unanimously that the progression criteria had been met.

## Discussion

### Principal Findings

#### Participant Overview

In a 2-armed, parallel-group fRCT, we assessed whether and how it would be possible to carry out a future definitive RCT of Wrapped. Our primary objectives were to estimate the rate of participant recruitment and follow-up. Over a 31-week period, 173 participants were recruited and provided a baseline chlamydia test result, representing 1.5% of the sampling pool. A valid chlamydia self-sample (primary outcome measure) was returned by 75.7% (131/173) at the final 12-month follow-up. Based on this information, 3574 participants, derived from a sampling pool of 238,266 service users, are required to power a future full trial (see Multimedia Appendix 5). Although this is a large sampling pool, approximately 585,000 16- to 24-year-olds use Preventx services each year (data provided in personal communication) making achievement of the required follow-up sample feasible.

#### Recruitment

Our analysis showed that all advertisement types appeared to work equally well. Although changing advertisement wording in the full trial may not therefore confer much benefit, increasing advertisement visibility and prominence may be useful strategies. With support of a user experience (UX) expert, we reviewed the recruitment journey and identified two changes that could increase recruitment. These included positioning the advertisement higher up on the Preventx “thank you” page (so that scrolling down was not required to view it) and using the power of the Preventx brand (trusted by our target population to process sensitive information) by using wording such as “we are partnering with Wrapped to...” These low-cost changes could have a sizeable impact on uptake, and we will seek to implement them in the full trial.

Imbalances were observed across demographic subgroups at baseline when comparing the intervention group with the control group. These were for sexual identity (a lower proportion of bisexual participants), age (a lower proportion of 16- to 19-year-olds and a higher proportion of 20- to 24-year-olds), and gender (a lower proportion of women and a higher proportion of men). For sexual identity and age, these imbalances are likely a product of our randomization strategy. To keep the total number of stratification categories to within reasonable limits, we did not stratify by age, and for sexual identity, the binary categorization of “heterosexual” versus “other” was used. This does not present a concern for a full trial, as balance would be expected to occur naturally given the large number of participants involved, thus negating the need for stratification altogether. The observed differences by gender are not a result of our stratification strategy but instead a result of selecting a subsample for analysis postrandomization (balance across groups for men and women at baseline was observed for the full sample; see full report provided to the funder [20]). Again, this does not present a concern for a full trial, as we would not be adopting this practice; randomization would occur once a participant’s STI status at baseline was known, and all randomized participants would be included in the analysis.

An observation that does have implications for a full trial is the level of chlamydia positivity at baseline (8 cases of 173 participants; 4.6%), which was lower than the national figure for users of web-based STI test settings (8.8%) at the time of data collection [30]. One explanation for this is the COVID-19 context of the study, which is known to have influenced the sexual behavior of young people in ways that are likely to have reduced STI risk [31,32]. This, however, does not explain the discrepancy in chlamydia positivity between our sample and national-level data from the same trial period. One possibility is that this reflects local differences in behavior or changes to service delivery in response to COVID-19 for areas participating in our study that are not evident in the national picture. Another possibility is that the discrepancy is unrelated to the COVID-19 pandemic and instead indicates a tendency for participation in this study by service users with lower sexual risk behavior. This is problematic because, if this estimated level of positivity is a true reflection of the sample likely to be recruited in the full trial, this threatens our sample size calculation (based on positivity being 10% in the control group; see Multimedia Appendix 5) and the external validity of the trial itself. Sexual health–related research is equivocal on whether recruited samples tend to exhibit higher or lower sexual risk behavior [33-35]. A recent longitudinal Dutch study, however, reported that baseline STI positivity was a predictor of nonparticipation [33]. Although we ought to be cautious in drawing too much inference from these data given the small-scale nature of the study and that chlamydia positivity was self-reported at baseline, it would be prudent when planning for a full trial to consider how to address disproportionate uptake of individuals by STI test outcome. Working with a PPI group, which includes young people who have experience with testing positive following online STI self-sampling, to review the recruitment process (particularly the wording of the study advertisement and participant information) will be an important aspect of this.

#### Retention and Data Completeness

The proportions of participants who provided a valid chlamydia self-sample were 75.1% (130/173) at M3 and 75.5% (131/173) at M12 (our primary outcome measure). This matches or exceeds response rates for the return of STI self-samples reported in other recent research, such as in the sexual health trial by Nicholas and colleagues [36] reporting 47.4% return by 16- to 20-year-olds at 3 months, the sexual health trial by Free and colleagues [37] reporting 74.8% return at 12 months by 16- to 24-year-olds, and a longitudinal study by van Wees and colleagues [33] reporting 75.7% return at 6 months by 18- to 24-year-olds. Although the overall level of retention was good, there was evidence of drop-out attrition, with return of a valid chlamydia self-samples at M12 being higher for participants in the intervention group (86%) than the control group (66%). This was unexpected given that meta-analyses of behavior change interventions, including for HIV prevention, have on average found retention to be slightly higher in the control group than in the intervention group [38,39]. The reason for this pattern of attrition is unknown. It may be that those in the intervention group, receiving free products from Wrapped, felt more of an obligation to complete follow-up assessments [40]. Alternatively, it may be that blinding was not entirely successful, with participants assigned to the control group feeling a sense of disappointment, which then led to drop-out [41]. A future trial should make it clear within the participant information that randomization is a feature of the study and that participants in both groups are equally valued, with full and complete data required from everyone if the study is to produce robust findings.

In this study, we also measured survey completeness, with response rates at all time points exceeding 90%. This aligns with recent trials of digital sexual health interventions, which have achieved 88% at the M1 survey follow-up [37] and 72% at the M3 survey follow-up [42]. Sustaining this level of response across all surveys through the M12 follow-up is particularly encouraging, given that trials involving online interventions are often vulnerable to high attrition rates [43,44]. Individual items required to assess primary and secondary outcomes for the main trial also achieved over 95% completion, with 65.9% of participants completing all 5 items required to measure chlamydia cumulative incidence. This reflects strong measure acceptability and likely stems from the extensive investment in the recruitment and retention strategy, as well as the iterative testing and refinement of measure delivery and completion in collaboration with our PPI group [19].

#### Differential Attrition by Participant Characteristics

The distribution of participants across IMD quintiles at M12 was compared with that within the sampling pool and at baseline to assess potential threats to internal and external validity in this respect. There was evidence of initial under-recruitment of participants in quintile 3 and of over-recruitment of participants in quintile 5 (least deprived), compounded by a degree of attrition for those in quintile 3 and strong retention for those in quintile 5. Efforts will need to be made in a future trial to achieve balance. Encouragingly, good representation of service users in deprivation quintile 1 (most deprived) was observed at both baseline and M12. However, given that trials often struggle to engage participants from more deprived backgrounds [45] and that caution should be taken not to overinterpret the findings given this study’s small overall sample size, future trial strategies should prioritize efforts to recruit and retain participants from deprivation quintiles 1 to 3. In a future trial, we would collaborate with a PPI group to explore what motivates young people from more deprived backgrounds to engage in research and to identify and address the barriers to their continued participation.

Comparison of M12 with baseline samples across demographic characteristics showed no evidence of differential attrition by gender, ethnicity, or sexual identity. Comparison of the M12 sample with the sampling pool, however, suggested that male participants had been slightly under-recruited and that Black participants had been slightly over-recruited. Any future trial ought to carefully monitor sample representativeness at baseline, with particular attention given to these characteristics and action taken to boost recruitment where needed. With regards to age, a higher proportion of participants aged 20 years to 24 years and a lower proportion of participants aged 16 years to 19 years were observed in the final sample relative to baseline. This pattern was also reflected when comparing the M12 sample with the sampling pool, suggesting that younger participants were initially under-recruited and were also more prone to drop-out. Lower participation among those younger than 20 years may stem from this age group being more likely to live at home with parents or carers and the concern that they may discover an STI self-sampling kit or intercept test result notifications [36,46]. This is partly supported by our analysis of adverse events, which showed that over one-half of the instances of the reported issue, “someone finding out I was testing for an STI when I didn’t want them to know,” were attributed to participants younger than 20 years, even though this age group comprised only 13% of the total sample. This was also, overall, the most reported adverse event, with only two instances of other types of events reported. In a full trial, it would be imperative to work with a PPI group, particularly younger members, to review barriers to study uptake and ongoing participation for younger people, so that strategies can be put in place to mitigate these. This could include, for example, providing additional reassurances about the discreet nature of STI self-sampling kits and SMS text message notifications and using postal lockers to avoid home delivery.

#### Contamination

Exposure to Wrapped was indicated by 43.3% of participants in the control group. Different routes to contamination were explored. We had some limited evidence of individuals joining the study having been told about it by an existing participant. We had no evidence, however, of participants, other than those assigned to the intervention condition, attempting to gain access to the site (owing to technological measures put in place to prevent access) or of log-in details being shared with others (from observation of analytics data). Although we cannot discount the possibility that contamination occurred through a participant in the intervention group physically sharing their screen with a participant in the control group, this alone would not account for the extent of contamination observed. Instead, the most likely explanation is that the question used to assess contamination, which asked control group participants whether they recognized images of the Wrapped website, was not reliable. This question must be revisited in any future trial such that participants are instead asked explicitly about access to intervention materials. There may also be merit in introducing measures to restrict the participation of individuals who know others already participating in the study.

#### Health Economic Assessment

Overall, processes put in place to capture outcome data were successful. This included processes for the EQ5D-5L and SF12 as well as costs associated with delivering the intervention and the control (usual care). This will enable a range of economic assessments to be conducted at full trial including a cost-effectiveness analysis and a cost-utility analysis as recommended by NICE [47,48]. The study did, however, highlight that the measures used in this study to capture the private sexual health costs of young people (eg, condoms, self-sampling kits) and their use of NHS health care resources could be improved. These measures need to be carefully codesigned with a PPI group ahead of any future trial to ensure that they result in more comprehensive data.

### Limitations

Recruitment to this study was slow. This was in part due to the disruption caused by the temporary and permanent losses of 4 of our 5 local authority sites. This took time to resolve and lengthened the recruitment period. To mitigate this, in a future full trial, we would ensure that all partner local authorities have a contract with Preventx for at least the duration of recruitment. We would also have plans in place to enable local authority partners to migrate between Preventx services with minimal disruption to recruitment. We would also hope to increase the overall proportion of the sampling pool recruited by increasing the visibility of our study advertisement. Although recruitment to any full trial at the rate observed in this study would still make the trial feasible, these changes have the potential to deliver desirable efficiency and cost reductions and therefore ought to be implemented.

As outlined in the previous paragraphs, the burden of STIs is known to be unequal among young people, with those who identify as Black or GBMSM or who live in areas of higher deprivation experiencing disproportionately high levels of diagnosis [1]. In preparation for a full trial, this study therefore examined whether the sample available for analysis represented the sampling pool across demographic characteristics, namely age, gender, IMD quintile, and ethnicity. In this study, we were, however, unable to do this for sexual identity, as these data were not provided by Preventx. For a full trial, obtaining sexual identity data for the sampling pool would be essential to enable comparisons with our sample to be made. Without this, we would be unable to comment fully on the external validity of our findings.

Our PPI group conducted a comprehensive review of all participant materials, focusing on their accessibility and inclusivity. Several revisions were made before the group provided final approval. However, we recognize that our PPI group lacked ethnic diversity, which may have limited our ability to enhance the cultural competence of the materials and procedures. Additionally, the PPI group did not include representatives aged 16 years to 19 years. In any future trial, a new PPI group with a more balanced composition would be established, ensuring good levels of representation of all groups disproportionately affected by STIs.

As discussed, we likely used an inadequate measure of contamination in this study. In trying to create an equivalent intervention experience for control group participants, we used the same website name (Wrapped) and branding as that used for the intervention website. The contamination measure then asked control group participants whether they recognized images from the intervention website (screenshots were shown). In any future trial, we would ensure that the two sites have distinct names and branding or change the nature of the contamination question.

### Recommendations for Designing an RCT

We have outlined in the previous sections the changes that are required should we proceed to a full trial of Wrapped. We also make recommendations here to others embarking on feasibility studies or full trials of behavioral or online interventions. First, as part of standard study risk assessments, we would recommend that teams carefully consider all assumptions regarding recruitment (eg, continued ability of sites to recruit) and develop contingency plans that can be swiftly enacted if necessary. Second, we would advise establishing a PPI group that closely mirrors the make-up of the sampling pool. If specific groups are identified as potentially harder to recruit or retain, we recommend prioritizing their inclusion within the PPI membership to ensure adequate representation. Last, we would recommend working with the PPI group (and a UX professional if available) to review the entire participant journey, removing any identified friction points that could have a negative impact on recruitment or retention.

### Conclusion

We have shown that a full trial of Wrapped is feasible. Our independent SSC and DMEC agreed and recommended progression to full trial. We have since been awarded funding from the National Institute for Health and Care Research (NIHR) to conduct that trial (NIHR portfolio number 157903). Learnings generated through this feasibility study will be applied to maximize successful trial conduct and delivery. If proven to be cost-effective, Wrapped has the potential to reduce STI diagnoses among young people, the negative health and quality of life consequences they experience as a result, and costs to the NHS and public health.

## Appendix

Multimedia Appendix 1 CONSORT 2010 checklist.

Multimedia Appendix 2 Schedule of vouchers.

Multimedia Appendix 3 Assignment of intervention components.

Multimedia Appendix 4 Supplementary results information.

Multimedia Appendix 5 Sample size calculation.

Multimedia Appendix 6 Cost and resource use.

Multimedia Appendix 7 Wrapped DMEC and SSC Progression Report

## Abbreviations

CONSORT: Consolidated Standards of Reporting Trials
DMEC: Data Monitoring and Ethics Committee
fRCT: feasibility randomized controlled trial
GBMSM: gay, bisexual, and other men who have sex with men
IMD: index of multiple deprivation
NICE: National Institute for Health and Care Excellence
NIHR: National Institute for Health and Care Research
PPI: Public and Patient Involvement
RCT: randomized controlled trial
REDCap: Research Electronic Data Capture
SF-12: Short Form 12
SSC: Study Steering Committee
STI: sexually transmitted infection
UX: user experience

## Data Availability

A copy of the raw anonymized research data, the syntaxes used for analysis, and the output generated are deposited within the University of Hertfordshire Research Archive (UHRA) [49] and made available indefinitely on an open access basis.
