# Supplementary material for: Intervention to Increase Condom Use Among Users of Sexually Transmitted Infection (STI) Self-Sampling Websites (Wrapped): Feasibility Randomized Controlled Trial
Source: J Med Internet Res. 2025 Aug 15;27:e71611. doi: 10.2196/71611 (PMC12397759; doi:10.2196/71611)
Supplement: Multimedia Appendix 2 [file jmir_v27i1e71611_app2.docx]

**Multimedia appendix 2 – Schedule of voucher payments**

| **Activity** | **First wave of recruitment**  **Weeks 1-7** | **Second wave of recruitment**  **Weeks 8-10** | **Third wave of recruitment**  **Weeks 11-31** |
| --- | --- | --- | --- |
| Month 0 (joining the study) | £5 | £10 | £15 |
| Self-report of baseline test result | £0 | £5 | £5 |
| Visiting the website | £0 | £5 | £5 |
| Month 3 test kit | £10 | £10 | £15 |
| Month 3 survey | £5 | £10 | £10 |
| Month 6 survey | £10 | £10 | £10 |
| Month 12 test kit | £20 | £20 | £25 |
| Month 12 survey | £15 | £15 | £15 |
| **Total** | **£65** | **£85** | **£100** |
