# Supplementary material for: Intervention to Increase Condom Use Among Users of Sexually Transmitted Infection (STI) Self-Sampling Websites (Wrapped): Feasibility Randomized Controlled Trial
Source: J Med Internet Res. 2025 Aug 15;27:e71611. doi: 10.2196/71611 (PMC12397759; doi:10.2196/71611)
Supplement: Multimedia Appendix 3 [file jmir_v27i1e71611_app3.docx]

**Multimedia 3 - Assignment of intervention components**

On first access to the Wrapped intervention, participants were presented with a series of ten statements, each of which represented a different barrier to condom use. Participants were asked to indicate which of these barriers applied to them clicking on a tick (yes) or a cross (no) next to each statement. Table 1 below shows the allocation of intervention components according to responses made. Where there is more than one statement allocated to a component, a participant only needed to tick one of these and that component would be made visible/accessible to them on the website.

Table 1 Intervention component assignment by tailoring statements

| **Component** | **Tailoring Statements** |
| --- | --- |
| Sample Pack | N/A  This component was offered to all participants regardless of responses to the tailoring questions |
| Condom Ordering Service | - “I can’t always get the type of condoms I want.” - “I find condoms expensive to buy.” - “I find buying condoms embarrassing.” |
| Condom Carrier | - “I don’t always have a condom on me when it’s needed.” |
| Condom Demo Video | - “I’m not always able to put a condom on with confidence and ease.” - “I find using condoms interrupts the flow of sex.” - “Condoms makes sex less enjoyable or pleasurable for me.” - “Condoms makes sex less enjoyable or pleasurable for the person I’m with.” |
| Discussing Condoms Videos | - “I find it awkward or difficult letting someone know that I want to use condoms.” - “I find using condoms interrupts the flow of sex.” |
| Real Life Videos | - “I find using condoms a turn-off.” - “I find using condoms interrupts the flow of sex.”      - “Condoms makes sex less enjoyable or pleasurable for me.” - “Condoms makes sex less enjoyable or pleasurable for the person I’m with.”   *NOTE: This component was for participants over the age of 18 only.* |
