# Supplementary material for: Intervention to Increase Condom Use Among Users of Sexually Transmitted Infection (STI) Self-Sampling Websites (Wrapped): Feasibility Randomized Controlled Trial
Source: J Med Internet Res. 2025 Aug 15;27:e71611. doi: 10.2196/71611 (PMC12397759; doi:10.2196/71611)
Supplement: Multimedia Appendix 4 [file jmir_v27i1e71611_app4.docx]

**Multimedia Appendix 4 – Results section supplementary information and tables**

Research Objective 4

Additional analysis was performed to compare the relative proportions of individuals in the sampling pool with participants at M12 across gender, ethnicity, and age (see table 1 below). Differences in the relative proportions were observed as follows. For gender, there appeared to be a slight under-representation of male participants at M12 compared to the sampling pool. For ethnicity, there appeared to be a slight over-representation of Black participants at M12 compared to the sampling pool. For age, participants were grouped as 16-19 years and 20-24 years as inspection across individual ages indicated differing trends across these broader categories and grouping aided making comparisons. Compared to those in the sampling pool, there appeared to be a lower relative proportion of 16–19-year-olds and a higher relative proportion of 20-24-year-olds at M12.

Table S1. Distribution of demographic characteristics amongst service users in the sampling pool and participants in the study at 12 months follow-up

|  | **Sampling pool**  **(n=11,413)** | **M12 follow-up**  **(n=131)** |
| --- | --- | --- |
|  | **N (%)** | **N (%)** |
| **Gender** | | |
| Female | 8057 (70.6) | 96 (73.3) |
| Male | 3305 (29.0) | 32 (24.4) |
| Other^a^ | 51 (0.4) | 3 (2.3) |
| **Ethnicity** | | |
| White | 11,413 (89.0) | 113 (86.3) |
| Mixed or multiple ethnic groups | 578 (5.1) | 4 (3.1) |
| Asian or Asian British | 254 (2.2) | 3 (2.3) |
| Black, African, Caribbean, or Black British | 276 (2.4) | 10 (7.6) |
| Other ethnic group | 142 (1.2) | 1 (0.8) |
| **Age** | | |
| 16-19 | 2855 (25.0) | 11 (8.4) |
| 20-24 | 8558 (75.0) | 120 (91.6) |

^a^Other includes trans-gender and non-binary/fluid gender (data on these gender categories not reported by Preventx and therefore unavailable for comparison purposes)

Research Objective 5

Table S2. Proportion of participants retained in the study at M12 by randomized group

| **Intervention** | **Control** | **Total** | | |
| --- | --- | --- | --- | --- |
| **n/N**  **(%)** | **n/N**  **(%)** | **n/N**  **(%)** | **95% CI** | |
|  |  |  | **Lower limit** | **Upper limit** |
| 72/84  (85.7) | 59/89  (66.3) | 131/173  (75.7) | 68.6 | 81.9 |

Table S3. Retention of participants by test result at baseline

| **Test result** | **Participants at baseline with each test result** | **Participants with each result at baseline who went on to provide a valid chlamydia self-sample at M12** | | |
| --- | --- | --- | --- | --- |
|  | **N** | **N (%)** | **Lower 95% Confidence limit** | **Upper 95% Confidence Limit** |
| Chlamydia Positive | 8 | 5  (62.5) | 24.5 | 91.5 |
| Chlamydia Negative | 165 | 126  (76.4) | 69.1 | 82.6 |
| Not reported | 57 | 20  (35.1) | 22.9 | 48.9 |

Note that all those who tested positive for chlamydia confirmed that they had taken the full course of treatment thus the sum of participants testing positive and negative at baseline is equal to the size of the sub-sample reported on in this paper (n=173). Also included is retention by those who did not report a result at baseline. This is markedly lower than for those reporting their test result thus further exemplifying the rationale for excluding this group at baseline in any future trial.

Note: Based on these findings, chlamydia positivity in our sample at baseline was 4.6% (8/173).

Research Objective 7

Table S4. Proportion of participants completing each study measure

| **Measure** | **Proportion of participants^a^ completing study measure n/N (%)** |
| --- | --- |
| M0 survey | 173/173 (100) |
| Self-reported result of chlamydia test | 173/173 (100) |
| M3 survey | 158/173 (91.3) |
| M6 survey ^b^ | 157/173 (90.8) |
| M12 survey | 157/171 (91.8) |
| Valid chlamydia sample at M3 | 130/173 (75.1) |
| Valid chlamydia sample at M12 | 131/171 (76.6) |

^a^ includes only participants invited to complete the measure; two participants withdrew after M6 and were therefore not invited to complete the M12 self-sample or survey

^b^ two participants completed a brief version of the survey over the telephone; these participants are included in the total number of completers

Research Objective 8

Note: there were no missing data for any of the items collecting demographic information as these items were set as mandatory fields in the baseline survey. Table 5 below displays the number and percentage of participants who completed each survey measure who had missing data on the remaining items.

Table S5. Percentage of missing data on measures of primary and secondary outcomes

| **Item** | **n/N (%) of participants^a^ with missing data** |
| --- | --- |
| **Chlamydia infection** |  |
| Self-report of any chlamydia infection (including whether treated, if applicable) within M0 survey | 0/173 (0) |
| Self-report of any chlamydia infection (including whether treated, if applicable) within M3 survey | 0/158 (0) |
| Self-report of any chlamydia infection (including whether treated, if applicable) within M6 survey^bc^ | 0/157 (0) |
| Self-report of any chlamydia infection (including whether treated, if applicable) within M12 survey | 0/157 (0) |
| **Condom use** |  |
| Self-report of condom use at M0 | 1/173 (0.6) |
| Self-report of condom use at M3 | 1/158 (0.6) |
| Self-report of condom use at M6^bd^ | 0/157 (0) |
| Self-report of condom use at M12 | 0/157 (0) |
| **Health-related quality of life (to inform a future economic evaluation)** |  |
| EQ5D-5L at M0 | 0/173 (0) |
| EQ5D-5L at M3 | 3/158 (1.9) |
| EQ5D-5L at M6^e^ | 2/157 (1.3) |
| EQ5D-5L at M12 | 2/157 (1.3) |
| SF12 at M0 | 2/173 (1.2) |
| SF12 at M3 | 3/158 (1.9) |
| SF12 at M6^f^ | 6/157 (3.8) |
| SF12 at M12 | 2/157 (1.3) |

^a^ includes only participants invited to complete each measure; two participants withdrew after M6 and were therefore not invited to complete the M12 self-sample or survey

^b^ two participants completed a brief version of the M6 survey over the telephone which included this item; these participants are included in the total number of completers

^c^ three participants completed this item but not the full M6 survey and are not counted as missing

^d^ two participants completed this item but not the full M6 survey and are not counted as missing

^e^ two participants completed this item but not the full M6 survey and are not counted as missing

^f^ one participant completed this item but not the full M6 survey and are not counted as missing

Research Objective 9

Note: We have presented data on the full sample (n=230) in Table VI.

Table S6. The proportion of Preventx users recruited to the study per recruitment message and total incentive offered

| **Advert number** | **Advert text** | **Length of time advert displayed (in weeks)** | **Incentive amount as communicated in advert and/or participant information** | **Percentage (n/N) of service users recruited following presentation of advert** |
| --- | --- | --- | --- | --- |
| 1 | Help us make a positive change to young people’s sexual health: make a difference by joining our study and get paid for your time! | 2 | £65 | 1.9 (19/954) |
| 2 | Take part in a study to improve sexual health and earn up to £65 in Amazon vouchers, find out how you can make a difference today! | 2 | £65 | 2.0 (19/941) |
| 3 | Your views are important: join our study to help improve sexual health for young people whilst being paid! | 2 | £65 | 1.8 (17/941) |
| 4 | Make a difference to improve sexual health for young people - join our study and earn up to £65 in Amazon vouchers for your time! | 1 | £65 | 3.5 (9/254) |
| 5 | Interested in sexual health research? Take part and get up to £85 in vouchers | 3 | £85 | 3.5 (20/577) |
| 6 | Help us make a positive change to young people’s sexual health: make a difference by joining our study and get up to £100 in vouchers. | 2 | £100 | 1.1 (5/460) |
| 7 | Take part in a study to improve sexual health and earn up to £100 in vouchers, find out how you can make a difference today | 2 | £100 | 1.6 (7/446) |
| 8 | Your views are important: join our study to help improve sexual health for young people  and get paid up to £100 in vouchers! | 2 | £100 | 2.6 (13/491) |
| 9 | Make a difference to improve sexual health for young people - join our study and earn up to £100 in vouchers for your time. | 3 | £100 | 2.0 (25/1282) |
| 10 | Want to make a difference to young people’s sexual health? Take part in our study and get up to £100 in vouchers for your time. | 2 | £100 | 1.6 (15/922) |
| 11 | We need your help! Take part in a study to improve young people’s sexual health and earn  up to £100 in vouchers for your time. | 2 | £100 | 0.9 (8/944) |
| 12 | Interested in sexual health research? Take part and get up to £100 in vouchers | 3 | £100 | 2.1 (28/1347) |
| 13 | *Shown to service users aged 20-24:*  Interested in sexual health research? Take part and get up to £100 in vouchers | 3 (plus 1 day) | £100 | 2.8 (39/1401) |
|  | *Shown to service users aged 16-19:*  Interested in Sexual Health Research and aged 16-19 years? Want to earn up to £100 in Amazon Vouchers? |  |  | 1.3 (6/ 453) |

Research Objective 10

Table S7. Number of patients reporting use of healthcare services (reported at M12)

| **Resource use** | **N Intervention Group** | **N Control group** | **Total** |
| --- | --- | --- | --- |
| Sexual Health clinic | 7 | 11 | 18 |
| GP consultation | 2 | 5 | 7 |
| GP nurse consultation | 2 | 2 | 4 |
| NHS walk in centre | 1 | 0 | 1 |
| NHS 111 calls | 1 | 0 | 1 |
| GP out of hours | 0 | 0 | 0 |
| Pharmacy | 1 | 1 | 2 |
| A&E | 0 | 0 | 0 |
| Gynaecology | 0 | 2 | 2 |
| other | 0 | 0 | 0 |
| Total number of participants reporting use of healthcare resources | 10 | 16 | 26 |
| Total number of participants completing this item at follow up | 96 | 95 | 191 |

Research Objective 12

Note: We have presented data on the full sample (n=230) in Table VIII.

Table S8. Frequency of adverse events reported along with any qualitative comments received about the event

| **Adverse event** | **Number of instances reported** | | **Qualitative comments** |
| --- | --- | --- | --- |
|  | **Intervention** | **Control** |  |
| Led to someone finding out I was having sex when I didn't want them to know | 1 | 0 | None |
| Led to someone finding out I was testing for an STI when I didn't want them to know | 5 | 4 | Email response on offer of support: “Its ok this hasn't caused me too much trouble, there is no need for any discussion, it wasn't a serious matter and there is no reason to change anything that you currently do”  Open-ended response in M12 survey: “It has made some uncomfortable conversations, but they have also been positive in showing that I am trying to be safe” |
| Led to an increase in my use of pornography | 0 | 1 | None |
| Other problem | 0 | 0 | None |
