# Supplementary material for: Intervention to Increase Condom Use Among Users of Sexually Transmitted Infection (STI) Self-Sampling Websites (Wrapped): Feasibility Randomized Controlled Trial
Source: J Med Internet Res. 2025 Aug 15;27:e71611. doi: 10.2196/71611 (PMC12397759; doi:10.2196/71611)
Supplement: Multimedia Appendix 5 [file jmir_v27i1e71611_app5.docx]

**Multimedia appendix 5 – Sample size calculation for full trial**

As outlined in research objective six, our original plan was to use estimates of chlamydia positivity in the intervention and control groups to inform the sample size calculation for a future full trial. However, with the approval of our SSC and DMEC, alternative sources were prioritized. This decision was based on concerns about the reliability of our estimates, given the small sample size (n=173) and the potential downward impact of the COVID-19 pandemic on population chlamydia positivity rates (see discussion). Instead, we used extensive consultation and document review to determine the necessary parameters for the sample size calculation.

Chlamydia positivity in the control group was estimated at 10%, based on 2023 UKHSA STI data for 16–24-year-olds testing for chlamydia via the internet [1]. To estimate the target positivity rate for the intervention group, we drew on several sources: (1) existing evidence of chlamydia positivity among young people in the general population (range: 4–6%; see[2–4]), (2) data showing a 6% positivity rate among young people tested via community-based GP practices [1], and (3) feedback from thirteen sexual health commissioners and service managers across five English regions, who agreed that a clinically meaningful reduction would fall between 4–6% for the intervention group, representing a 2–4% difference between groups.

Based on advice from an independent expert group, we determined that the trial should be powered to detect a 3.5% difference in positivity rates (10% in the control group vs. 6.5% in the intervention group). Using G*Power, we calculated that 2,706 participants would be required to detect this difference, necessitating a total trial sample of 3,574 participants (accounting for 75.7% retention; 2,706/0.757). Assuming a recruitment rate of 1.5%, a sampling pool of 238,266 users would be required to meet this target (3,574/0.015).

The number of 16–24-year-olds using Preventx for chlamydia self-sampling varies annually, but recent data indicates a pool of approximately 585,000 users (personal communication). This suggests that recruitment over a 12-month period for a full trial is feasible.

**References**

1. UK Health Security Agency. National chlamydia screening programme (NCSP): annual data. 2024. Available from: https://www.gov.uk/government/statistics/national-chlamydia-screening-programme-ncsp-data-tables [accessed Dec 16, 2024]

2. Bracebridge S, Bachmann MO, Ramkhelawon K, Woolnough A. Evaluation of a systematic postal screening and treatment service for genital Chlamydia trachomatis, with remote clinic access via the internet: a cross-sectional study, East of England. Sexually transmitted infections London: BMJ Publishing Group Ltd; 2012;88(5):375–381. doi: 10.1136/sextrans-2011-050267

3. Oakeshott P, Kerry-Barnard S, Fleming C, Phillips R, Drennan VM, Adams EJ, Majewska W, Harding-Esch EM, Cousins EC, Planche T, Green A, Bartholomew RI, Sadiq ST, Reid F. ‘Test n Treat’ (TnT): a cluster randomized feasibility trial of on-site rapid Chlamydia trachomatis tests and treatment in ethnically diverse, sexually active teenagers attending technical colleges. Clinical microbiology and infection England: Elsevier Ltd; 2019;25(7):865–871. doi: 10.1016/j.cmi.2018.10.019

4. Kalwij S, French S, Mugezi R, Baraitser P. Using educational outreach and a financial incentive to increase general practices contribution to chlamydia screening in South-East London 2003-2011. BMC public health England: BioMed Central Ltd; 2012;12(1):802–802. doi: 10.1186/1471-2458-12-802
