# Supplementary material for: Intervention to Increase Condom Use Among Users of Sexually Transmitted Infection (STI) Self-Sampling Websites (Wrapped): Feasibility Randomized Controlled Trial
Source: J Med Internet Res. 2025 Aug 15;27:e71611. doi: 10.2196/71611 (PMC12397759; doi:10.2196/71611)
Supplement: Multimedia Appendix 6 [file jmir_v27i1e71611_app6.docx]

**Multimedia Appendix 6: Calculation of intervention and recourse use costs**

**Fixed intervention costs**

Fixed costs were estimated for maintaining and updating the content of both the Wrapped and control websites. Using methods adopted previously in similar studies [1], these were estimated at £2 per user for the Wrapped website and £1 per user for the control website. This assumed that both websites were widely used across local authorities.

**Variable intervention costs**

Variable costs for the Wrapped website were associated with the cost of the condom sample packs, condom carriers, and condom ordering service, including that of packaging and distribution. Costs associated with these three items are provided in tables 1 to 3 below (with the estimated cost per participant based on orders made by participants in the intervention condition, full sample). There were no variable costs for the control website.

Table 1 Cost details for sample packs

| **Item** | **Unit cost (£^a^)** |
| --- | --- |
| **Condom sample pack- packaging** | |
| Condom sample pack – box | 1.41 |
| Condom sample pack- leaflet printing | 0.94 |
| Condom sample pack- - inlay printing | 0.34 |
| Condom sample pack- condom menu sheet printing | 0.07 |
| Total cost of packaging | 2.76 |
| **Condom sample pack- contents** | |
| Durex Pleasure Me | 0.53 |
| Durex Thin Feel | 0.43 |
| EXS Air Thin | 0.10 |
| EXS Extra Large | 0.08 |
| EXS Snug Fit | 0.08 |
| MyONE Perfect Fit | 1.17 |
| Pasante Cooling | 0.09 |
| Pasante Sensitive | 0.08 |
| Pasante Warming | 0.09 |
| Skyn Extra Lubricated | 0.46 |
| Skyn Intense Feel | 0.50 |
| Skyn Original | 0.29 |
| Lube- silky TLC | 0.08 |
| Lube – light /gentle | 0.08 |
| Total condom and lube costs | 4.06 |
| **Other Costs** | |
| Postage bag | 0.13 |
| Address sticky label | 0.03 |
| Postage costs | 1.53 |
| Thank-you card | 0.07 |
| Assembly costs^b^ | 0.36 |
| Total Other Costs | 2.12 |
| **Grand Totals** | |
| **Total cost of one sample pack**^c^ | £8.94 |
| Total orders of sample pack | 48 |
| Total cost | £429.12 |
| **Cost per participant in the intervention arm** | **£3.73** |

^a^Costs are £UK (2021/22) and include VAT where applicable

^b^Assumes packs are assembled by administrative assistant and this takes 2 mins (based on trial data and assuming £10.90 hourly rate)

^c^Based on costs recorded in the trial. Assumes that unused sample packs can be utilised in other ways

Table 2 Cost details for condom carrier

| **Item: Condom Carrier** | **Unit cost (including VAT & shipping costs) (£^a^)** |
| --- | --- |
| Condom carrier | 3.17 |
| Condom | 0.08 |
| Postage bag | 0.10 |
| Thank you card | 0.07 |
| Address sticky label | 0.03 |
| Postage costs | 0.88 |
| Assembly costs^b^ | 0.36 |
| **Grand Totals** | |
| **Total cost of one carrier**^c^ | 4.69 |
| Total orders of condom carriers | 31 |
| Total cost | 145.39 |
| **Cost per participant in intervention arm** | 1.26 |

^a^Costs are £UK (2021/22) and include VAT where applicable

^b^Assumes packs are assembled by administrative assistant and this takes 2 mins (based on trial data and assuming £10.90 hourly rate)

^c^Based on costs recorded in the trial. Assumes that unused carriers can be utilised in other ways.

Table 3 Condom ordering service costs (over the trial period)

| **Item** | **Unit cost (£^a^)** | **N** | **Total cost (£^a^)** |
| --- | --- | --- | --- |
| **Condoms** | | | |
| Durex Pleasure Me | 0.53 | 222 | 117.66 |
| Durex Thin Feel | 0.43 | 234 | 100.62 |
| EXS Air Thin | 0.1 | 60 | 6.00 |
| EXS Extra Large | 0.08 | 48 | 3.84 |
| EXS Snug Fit | 0.08 | 12 | 0.96 |
| MyONE Perfect Fit | 1.17 | 78 | 91.26 |
| Pasante Cooling | 0.09 | 30 | 2.70 |
| Pasante Sensitive | 0.08 | 60 | 4.80 |
| Pasante Warming | 0.09 | 42 | 3.78 |
| Skyn Extra Lubricated | 0.46 | 123 | 56.58 |
| Skyn Intense Feel | 0.50 | 243 | 121.50 |
| Skyn Original | 0.29 | 129 | 37.41 |
| **Total cost of condoms** |  |  | **547.11** |
| **Other Costs** | | | |
| Text messages/ email reminders | 0.01 | 37 | 0.37 |
| Instruction Sheet | 0.60 | 115 | 69.00 |
| Thank you card | 0.07 | 115 | 8.05 |
| Postage bag | 0.10 | 115 | 11.50 |
| Address sticky label | 0.03 | 115 | 3.45 |
| Postage costs | 0.96 | 115 | 110.40 |
| Assembly costs^b^ | 0.36 | 115 | 41.40 |
| **Total other costs** |  |  | **244.17** |
| **Grand totals** | | | |
| Total costs for condom ordering service (condoms and other costs) ^c,d^ |  |  | 791.28 |
| Number of people in intervention arm |  | 115 |  |
| **Cost per participant in the intervention arm** |  |  | **6.88** |

^a^Costs are £UK (2021/22) and include VAT where applicable

^b^Assumes orders are assembled by administrative assistant and this takes 2 mins (based on trial data and assuming £10.90 hourly rate)

^c^Based on costs recorded in the trial. Assumes that unused condoms/other materials can be utilised in other ways.

^d^This is the total cost for 115 orders placed by 37 participants

**Resource use costs**

The table below gives an illustrative summary of the resource use costs collected as part of the feasibility study.

Table 4 Illustrative summary of resource use costs collected

| **Type of Resource use (based on data reported at 12 months)** | **Total cost per participant - Intervention (£^a^)** | **Total cost per participant – Control (£^a^)** |
| --- | --- | --- |
| Healthcare service resource use | 28.63 | 34.67 |
| Other NHS resource use – average costs per participant | 34.63 | 25.29 |
| Resource use via schools, colleges, universities | 0.47 | 0 |
| Private costs | 12.43 | 17.47 |
| **Average cost per participant** | **76.16** | **77.43** |

^a^Costs are presented in UK£ 2021/22
