# Supplementary material for: Intervention to Increase Condom Use Among Users of Sexually Transmitted Infection (STI) Self-Sampling Websites (Wrapped): Feasibility Randomized Controlled Trial
Source: J Med Internet Res. 2025 Aug 15;27:e71611. doi: 10.2196/71611 (PMC12397759; doi:10.2196/71611)
Supplement: Multimedia Appendix 7 [file jmir_v27i1e71611_app7.docx]

 
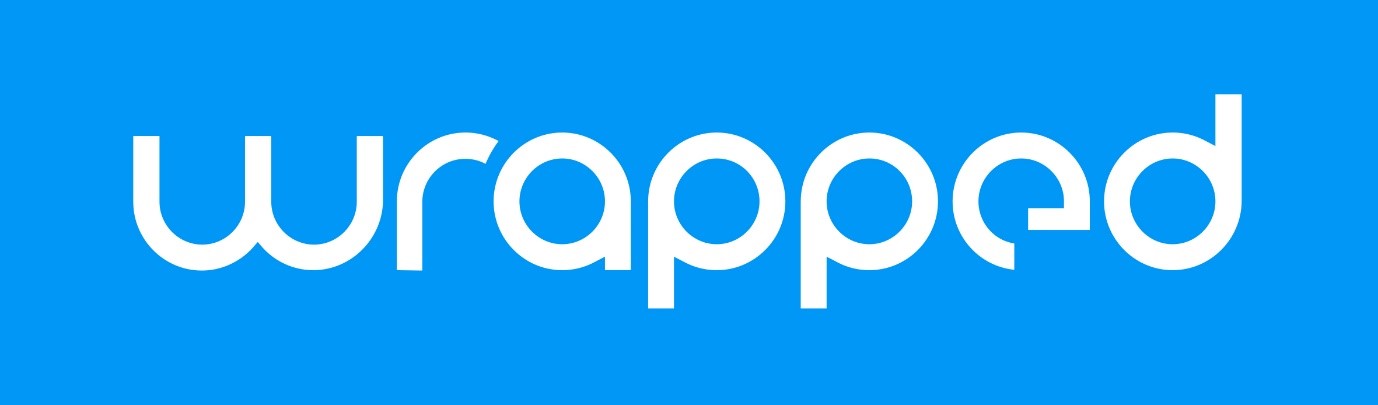


Data Monitoring and Ethics Committee

and Study Steering Committee

Progression Report

Document Version 2.0

March 2023

**Study/Trial Full Title:**An interactive digital behaviour change intervention (Wrapped) to decrease incidence of sexually transmitted infections (STIs) amongst users of STI self-sampling websites: A randomised controlled feasibility trial (PHR NIHR128148)

**Short title:** Wrapped feasibility trial

**Project Start/End dates:** 1^st^ May 2020 to 30 April 2023 (36 months)

**REC Reference:**20/EM/0275

**ISRCTN Number**: [ID ISRCTN17478654](http://www.isrctn.com/ISRCTN17478654)

**Principal Investigator**: Dr Katie Newby

**Sponsor**: University of Hertfordshire

**VERSION HISTORY**

| Date | Document Version | Revision history | Author |
| --- | --- | --- | --- |
| 08/03/2023 | 2.0 | First draft sent to SSC | KN |
|  |  |  |  |

**Abbreviations**

| DMEC | Data Monitoring and Ethics Committee |
| --- | --- |
| fRCT | Feasibility Randomised Controlled Trial |
| PI | Principal Investigator |
| RCT | Randomised Controlled Trial |
| SSC | Study Steering Committee |
| TMG | Trial Management Group |

Contents

[Progression Criteria 1 3](#_Toc129178139)

[Progression criteria 2 6](#_Toc129178147)

[Progression criteria 3 7](#_Toc129178148)

[Progression criteria 4 8](#_Toc129178149)

[References 9](#_Toc129178150)

[Appendix 10](#_Toc129178151)

**Progression Criteria**

The progression criteria, as amended and approved by HRA and the funder in June 2022, are as follows:

- 1. The proportion of Preventx users recruited to the feasibility trial is sufficient to obtain the sample size required for the definitive RCT
  2. 60% of participants (those randomised and known to be absent of chlamydia at baseline) followed-up for the definitive RCT primary outcome measure at 12 months
  3. IMD quintile distribution for the final sample is comparable to that of individuals in the sampling pool (Preventx users in the partner local authority areas)
  4. Adverse events are judged as sufficiently infrequent and/or serious to cause concern

The protocol stated that the progression decision was to be determined by the study’s independent Data Monitoring and Ethics Committee (DMEC) and Study Steering Group (SSG), based on achievement of the above criteria, or convincing evidence that any one criterion was amenable to sufficient improvement.

# Progression Criteria 1

| The proportion of Preventx users recruited to the feasibility trial is sufficient to obtain the sample size required for the definitive RCT |
| --- |

There are several key pieces of information regarding the definitive trial that are required to make this decision:

1. Size of the sampling pool available to recruit from
2. Estimated proportion of individuals that will be recruited from the sampling pool
3. The number of participants required to sufficiently power analysis of the primary outcome

## Size of the sampling pool

In our fRCT, we recruited participants from one online STI self-sampling provider (Preventx). Preventx have agreed to continue supporting the study should we move to full trial. Preventx operates across approximately 70 local authorities in England and distributes over 1.3 million STI self-sampling kits per year. The size of the sampling pool available to us at main trial will however depend on the number of commissioning local authorities and their partner trusts who are willing to support the study. To provide an indication of the willingness of areas to agree to this we have, together with Preventx, approached ten areas (representing variation in the demography of the population and urbanisation/rurality) to invite expressions of interest. So far, six of these areas have agreed to partner with us and we are in discussions with the remaining five (see Appendix A for details of the estimated number of test kits that would be ordered per 12-month and per 18-month periods within each of these areas). We will continue with these discussions and aim to gain expressions of interest from as many areas as required to obtain the sample size required at full trial. Preventx will support us in this process and can identify further areas to approach if required.

## Estimated proportion of individuals that will be recruited from the sampling pool

In our fRCT, an individual was randomised on completion of the baseline survey. For any a future trial, as previously agreed, we will only randomise participants whose baseline chlamydia test result is known (either via self-report as in the fRCT, or preferably via objective data provided by Preventx; participants who test positive will also be required to self-report that they have taken the prescribed treatment).

Of our 230 participants, 173 (75.2% of the full sample) self-reported their STI test result (with all those testing positive also reporting having received full treatment). With this additional criterion applied at full trial, we would therefore expect 1.5% of the sampling pool to be randomised (173/11,413).

Note: we have compared the demographic characteristics of the sample at baseline with those of the full sample and this sub-sample (herein referred to as the ‘restricted sample’) at 12 months (those who returned a valid chlamydia self-sample) - see appendix B for bar charts displaying the proportions and 95% confidence intervals (CIs)). Across all demographic characteristics (i.e. age, ethnicity, gender, index of multiple deprivation (IMD) quintile, and sexual identity), CIs overlapped across the baseline, full and restricted samples, indicating that using the restricted sample at full trial would not bias the sample available for analysis.

## The number of participants required to sufficiently power the primary analysis

To establish the sample size required at main trial, we first gathered information to inform a) the level of chlamydia positivity expected in the control group, and b) the level of positivity in the intervention group that we should seek to detect. This information, along with a range of sample size calculations, was presented to an independent expert group who advised the team.

### Level of positivity in control group

Evidence from feasibility studies can be used as an indication of the event rate in the control group at main trial. This in turn can then be used to inform the sample size calculation. Within our fRCT, positivity in the control group at month 12 was 2.6% (3/115). Given the low sample size, we decided to instead base the expected level of chlamydia positivity in the control group on national data.

Table 1 below (reproduced UKHSA report on [STIs and chlamydia screening, 2021](https://www.gov.uk/government/statistics/sexually-transmitted-infections-stis-annual-data-tables/sexually-transmitted-infections-and-screening-for-chlamydia-in-england-2021-report#national-chlamydia-screening-programme)) presents data on chlamydia positivity (15-24 year olds) by service type for 2021 (the most recent year there is full data for). Chlamydia positivity for internet testing is 8.4%.

TABLE 1 Chlamydia test positivity among 15- to 24-year-olds by test setting, 2020 to 2021, England


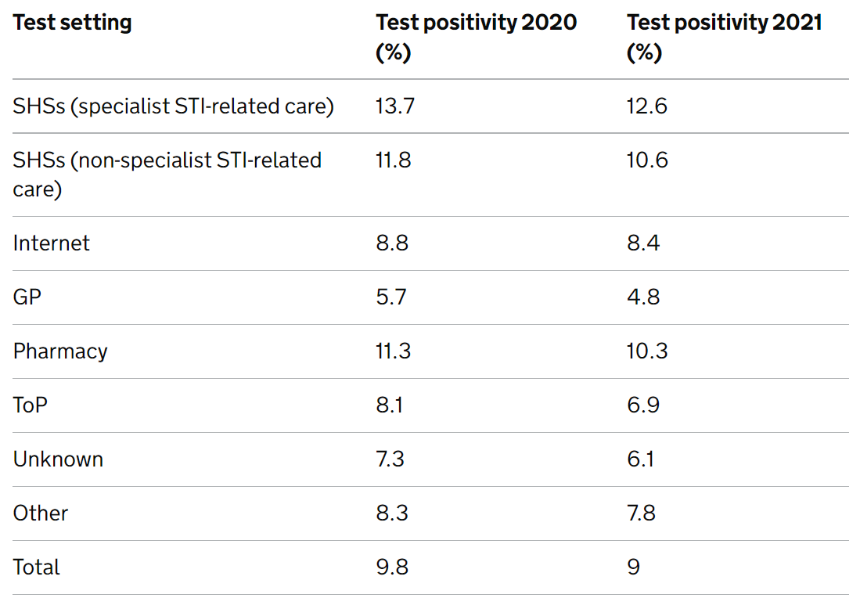


### The level of positivity in the intervention group that we should seek to detect

Data from multiple sources has been gathered to help us determine this.

#### Opinion of trusts/LAs on what would be a meaningful reduction in chlamydia positivity

Over the last four months we have interviewed sexual health commissioners (local authority based) and sexual health service managers (trust based) from all five areas involved in our fRCT. At each of these meetings we asked what they would consider to be a meaningful difference in chlamydia positivity between the trial arms; assuming 8% chlamydia positivity in the control arm. Appendix C presents the collective views of these colleagues. In summary, a difference of between 2-4 percentage points between the groups would be considered meaningful (i.e. between 4-6% in the intervention arm).

#### Evidence from the literature on what we might expect the effect of a behavioural intervention to be on a measure of chlamydia positivity

Following a rapid review we only found two relevant studies (1,2) however both were feasibility trials and therefore not informative.

#### Benchmarking against other groups

To provide a benchmark level of positivity that could be realistically aimed for in our intervention arm, our rapid review also searched for studies that provided an indication of chlamydia prevalence amongst young people in the general population:

Bracebridge et al 2012 (3): sent STI self-sampling pack to all 18-24 year olds in North East Essex PCT: ‘Of 3431 registered individuals tested, 152 (**4.4%**, 95% CI 3.8 to 5.2) had positive chlamydia test results, none of whom were symptomatic’.

Oakeshott et al 2019 (4): participants from 6 technical colleges (16-24 years) in London; found baseline chlamydia to be positivity of **5.1%** (3/59; 1.1-14.2%).

Kalwij et al 2012 (5) reported positivity rates of GP practice patients (16-24 year olds) in south-east London: ‘In Lambeth, positivity rate in 2010/11 was **5.8%** in men and **6.0%** in women. In Southwark positivity rate was **3.9%** in men and **5.3%** in women’.

A further benchmark that could be aimed for is the level of chlamydia positivity amongst young people accessing testing via GP practices (4.8%; see table above).

#### Expert opinion

An independent expert group was convened to advise the team consisting of the following individuals:

| Dr John Saunders | Clinical champion, National chlamydia screening programme, UK Health Security Agency; Sexual Health and HIV consultant, Central and North West London NHS Foundation Trust; Honorary senior lecturer, UCL |
| --- | --- |
| Kate Folkard | Public Health consultant; Head of Programme Delivery and Service Improvement (STIs, HIV, Hepatitis and Blood Safety), UK Health Security Agency |
| Rob  Bacon | Health Improvement Lead: Sexual Health, Hertfordshire County Council |

This group was asked to consider the above evidence. Following discussion, the group advised that we should be most strongly led by what commissioners felt was meaningful.

### Sample size calculation

The team discussed the above evidence and considered the advice of the independent expert group. A decision was made to power the study to detect a difference of 3% (i.e. 8% in control arm and 5% in intervention arm) based on:

- 5% positivity falling within the range identified as meaningful by sexual health commissioners/managers within our partner areas
- 5% broadly reflecting the levels of chlamydia observed amongst young people in lower risk groups i.e. general population and those testing via GP services (where positivity lowest)

A power calculation to estimate the sample size required to detect a difference of 3% was run using G*Power. Appendix D presents the result of this calculation.

### Summary

To detect a difference between 8% to 5%, a sample size of 2,966 participants is required.

Allowing for an estimated 75.7% of participants providing a valid chlamydia self-sample at month 12^[[1]](#footnote-2)^, the sample size required at full trial would be 3,918 participants (2,996/0.757= 3918).

Given that we estimate recruiting 1.5% of service users, a sampling pool of 261,200 would be required (3918/0.015). We propose recruiting over an 18-month period to maximise the size of the available sampling pool (although this may not be necessary, especially if we can secure commitment from sexual health London) and will continue to work with Preventx to secure further expressions of interest.

*Addendum (Janaury 2025): Since this report was produced, national levels of chlamydia positivity across test settings (i.e. internet and GP) have changed. The sample size calculation reported in the paper reflects the most recent data available at the time of writing and therefore supersedes that presented above (albeit the rationale remains consistent).*

# Progression criteria 2

| 60% of participants (those randomised and known to be absent of chlamydia at baseline) followed-up for the definitive RCT primary outcome measure at 12 months |
| --- |

TABLE 2 The proportion of participants (those randomised and known to be absent of chlamydia at baseline) who provided a valid chlamydia self-sample at month 12 by group

|  | Total |
| --- | --- |
| Total number of participants in the sample | 173 |
| Number of who provided a valid chlamydia self-sample at month 12 | 131 |
| Percentage | **75.7** |

# Progression criteria 3

| IMD quintile distribution for the final sample is comparable to that of individuals in the sampling pool (Preventx users in the partner local authority areas) |
| --- |

To identify whether the level of deprivation of participants in the final sample at 12 months (determined as those who returned an STI self-sampling kit at M12) was representative of service users in the wider sampling pool, the proportion (and 95% confidence intervals) of individuals in Index of Multiple Deprivation (IMD) quintiles one (most deprived) to five (least deprived) in each population were compared. Data for participants in both the full and restricted samples was analysed. This data is presented in tabular (see appendix E) and visual (figure 1 below) formats.

FIGURE 1 Bar chart (with 95% confidence intervals) displaying the proportion of individuals by IMD quintile within the sampling pool, the full sample at M12, and the restricted sample at M12

This data indicated that the M12 restricted sample was representative of the sampling pool across all IMD quintiles.

# Progression criteria 4

| Adverse events are judged as sufficiently infrequent and/or serious to cause concern |
| --- |

Within all follow-up surveys, participants were asked to self-report any adverse events related to their participation in the study. There were four fixed response items, three for anticipated adverse events, and one for ‘other problem’ which if endorsed routed participants to an open-ended question asking for further information.

Table 3 below presents the number of reported instances each of the adverse event types, along with any qualitative comments received from participants via email or survey, recorded across all time points. The majority of adverse events concerned disclosure of STI testing. One individual reported that participation in the study had led to an increased their use of pornography. This was unexpected given that this event was anticipated as a possible consequence of engagement with the intervention material and this participant was allocated to the control group. This individual was contacted and asked if they would be willing to provide further information, but none was received.

TABLE 3 Frequency of adverse events reported along with any qualitative comments received about the event

| **Adverse event** | **Number of instances reported** | **Qualitative comments** |
| --- | --- | --- |
| Led to someone finding out I was having sex when I didn't want them to know | 1 | None |
| Led to someone finding out I was testing for an STI when I didn't want them to know | 9 | Email response on offer of support: “Its ok this hasn't caused me too much trouble, there is no need for any discussion, it wasn't a serious matter and there is no reason to change anything that you currently do”  Open-ended response in M12 survey: “It has made some uncomfortable conversations, but they have also been positive in showing that I am trying to be safe” |
| Led to an increase in my use of pornography | 1 | None |
| Other problem | 0 | None |

# References

1. Free C, McCarthy O, French RS, Wellings K, Michie S, Roberts I, et al. Can text messages increase safer sex behaviours in young people? Intervention development and pilot randomised controlled trial. Health Technol Assess. 2016 Jul;20(57):1–82.

2. Bailey JV, Webster R, Hunter R, Griffin M, Freemantle N, Rait G, et al. The men’s safer sex project: Intervention development and feasibility randomized controlled trial of an interactive digital intervention to increase condom use in men. Health technology assessment (Winchester, England). 2016;20(91):1–152.

3. Bracebridge S, Bachmann MO, Ramkhelawon K, Woolnough A. Evaluation of a systematic postal screening and treatment service for genital Chlamydia trachomatis, with remote clinic access via the internet: a cross-sectional study, East of England. Sexually transmitted infections. 2012;88(5):375–81.

4. Oakeshott P, Kerry-Barnard S, Fleming C, Phillips R, Drennan VM, Adams EJ, et al. ‘Test n Treat’ (TnT): a cluster randomized feasibility trial of on-site rapid Chlamydia trachomatis tests and treatment in ethnically diverse, sexually active teenagers attending technical colleges. Clinical microbiology and infection. 2019;25(7):865–71.

5. Kalwij S, French S, Mugezi R, Baraitser P. Using educational outreach and a financial incentive to increase general practices contribution to chlamydia screening in South-East London 2003-2011. BMC public health. 2012;12(1):802–802.

# Appendix

## Appendix A

Areas approached to ascertain interest in participating at full trial, along with information on the estimated number of STI self-sampling kits ordered per area (based on most recent data)

| **Trust/local authority** | **Estimated number of STI self-sampling kits ordered per area** | | **Expression of interest?** |
| --- | --- | --- | --- |
|  | **12-months** | **18-months** |  |
| [Names of areas redacted due to commercial sensitivity] | 45,000 | 67,000 |  |
|  | 12,000 | 18,000 |  |
|  | 12,000 | 18,000 | Yes |
|  | 570,000 | 855,000 |  |
|  | 9,000 | 13,500 | Yes |
|  | 15,000 | 22,500 |  |
|  | 45,000 | 67,500 | Yes |
|  | 10,000 | 15,000 | Yes |
|  | 9,000 | 13,500 | Yes |
|  | 2,000 | 3,000 | Yes |
| **Total** | **729,000** | **1,093,5000** |  |

For areas that have so far submitted expressions of interest, the sampling pool is 87,000 (130,500 over 18 months). If any of the above do not wish to participate, there are other areas that can be approached.

## Appendix B

Bar charts (with 95% confidence intervals) displaying the proportion of individuals at baseline, the full sample at M12, and the restricted sample at M12 across measured demographic characteristics.

Age (years)

Gender

IMD decile

Ethnicity

Sexual identity

## Appendix C

Views of colleagues in partner trusts/public health departments on what would be considered a meaningful reduction in chlamydia positivity

| **Area** | **Meeting number** | **Contributors** | **Meaningful reduction** |
| --- | --- | --- | --- |
| East Sussex | 1 | Strategic Commissioning Manager - Sexual Health, East Sussex County Council    Service Manager – Sexual health, East Sussex Healthcare NHS Trust | **50%** |
| Northamptonshire | 1 | Commissioning Manager, Public Health Northamptonshire, North Northamptonshire Council | **50%** |
|  | 2 | Sexual health consultant, Northamptonshire Healthcare Foundation Trust, NHFT | **25%** |
| Kingston | 1 | Service Development & Commissioning Lead Communities, Royal Borough of Kingston upon Thames    Public Health Commissioning Support Officer, Royal Borough of Kingston upon Thames | **25%**    ‘if you can demonstrate a reduction in health inequalities, perhaps - they might not have to be such a big reduction, but if you can demonstrate a reduction in our more harder to reach groups … even if their reduction is not as big in those groups, that would still be quite important for us’ |
|  | 2 | Service Lead for Contraception & Sexual Health (CASH), Your Healthcare CIC | **37.5% (8% to 5% positivity)**    ‘Anything less than 8% (positivity) is good, but I mean if you were looking at a number then, you know, maybe 5%... but you know from my perspective, any number less than the 8% is beneficial’ |
| Somerset | 1 | Health Promotion Manager (Sexual Health), Somerset County Council    Specialist nurse advisor, SWISH  Specialist nurse advisor, SWISH | **50%**    ‘I think we'd have to see at least a 50% reduction’ |
| Warwickshire | 1 | Lead Commissioner Lifestyle and Prevention, Warwickshire County Council    Sexual Health Commissioner, Lifestyle and Prevention, Warwickshire County Council  Service Manager, Integrated Sexual Health Service, George Elliot Hospital NHS Trust | **25%**    ‘So maybe I would say 1/4 would be interesting… because I think 1/2 is too ambitious’ |

## Appendix D

Sample size calculation for 8% to 5%


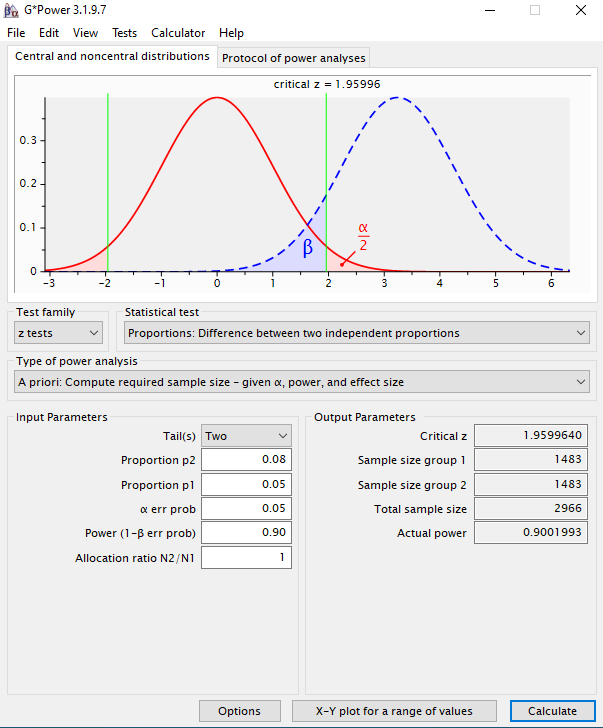


## Appendix E

Distribution of IMD quintiles amongst service users in the sampling pool and participants in the study at M12 follow-up (full and restricted samples)

| **IMD Quintile** | **Sampling Pool**  **(n=11,413)** | | | **M12 follow-up –**  **full sample**  **(n=151)** | | | **M12 follow-up – restricted sample (n=131)** | | |
| --- | --- | --- | --- | --- | --- | --- | --- | --- | --- |
|  | **N (%)** | **95% Confidence Interval** | | **N (%)** | **95% Confidence Interval** | | **N (%)** | **95% Confidence Interval** | |
|  |  | **Lower limit** | **Upper limit** |  | **Lower limit** | **Upper limit** |  | **Lower limit** | **Upper limit** |
| 1 (most deprived) | 1418 (12.4) | 11.8 | 13.0 | 24 (15.7) | 10.5 | 22.7 | 21 (16.0) | 10.2 | 23.5 |
| 2 | 2136 (18.7) | 18.0 | 19.4 | 22 (14.6) | 9.4 | 21.2 | 19 (14.5) | 9.0 | 21.7 |
| 3 | 3110 (27.2) | 26.4 | 28.1 | 31 (20.5) | 14.4 | 27.9 | 29 (22.1) | 15.4 | 30.2 |
| 4 | 2770 (24.3) | 23.5 | 25.1 | 36 (23.8) | 17.3 | 31.5 | 32 (24.4) | 17.4 | 32.7 |
| 5 (least deprived) | 1979 (17.3) | 16.6 | 18.0 | 38 (25.2) | 18.5 | 32.9 | 30 (22.9) | 16.0 | 31.1 |

1. This is the response rate for the restricted sample (i.e. those who reported their chlamydia test result at baseline). The response rate for the full sample is 65.7%. [↑](#footnote-ref-2)
